# Supplementary material for: Probabilistic classification of late treatment failure in uncomplicated falciparum malaria
Source: Nat Commun. 2025 Nov 10;16:9880. doi: 10.1038/s41467-025-64830-z (PMC12603158; doi:10.1038/s41467-025-64830-z)
Supplement: Supplementary file 1 — Supplementary Information [file 41467_2025_64830_MOESM1_ESM.pdf]

Supplementary Information for

Probabilistic classification of late treatment failure in uncomplicated falciparum malaria

Somya Mehra, Aimee R Taylor, Mallika Imwong, Nicholas J White, James A Watson

Contents

1 Derivation of model likelihood **1**

1.1 A locus-wise model predicated on allele frequencies 2

1.2 Modelling allele frequencies 5

1.2.1 Allele frequencies for population I 5

1.2.2 Allele frequencies for population C 5

1.2.3 A locus-wise model predicated on allele counts 6

1.3 Moments of allele counts 7

1.3.1 Per-sample allele counts 9

1.3.2 Pooled-sample allele counts 10

2 Validation against simulated data **11**

3 Empirical data: a re-analysis of Dimbu *et al.* [9] **14**

3.1 Within-host diversity 14

3.2 Sensitivity of PfRecur to user-specified parameters 14

3.2.1 Classification of recurrences in Dimbu *et al.* [9] 14

3.2.2 False positive recrudescence rates 21

3.3 Comparison against CDC model 21

3.3.1 Efficacy estimates 21

4 Comparison against CDC model structure [12] **25**

1 Derivation of model likelihood

We model a sample r from a recurrent infection as a mixture of clones drawn from two populations: population C comprising clones in the paired baseline sample b (this represents reCrudescence); and population I, reflecting the contemporaneous at large but approximated by the baseline population for which baseline samples $1, \ldots, n$ (excluding the baseline sample b, which is paired with r) constitute a finite sample (this represents reInfection).

Under our model, the probability that a given clone in sample r exhibits allele α is equated to the frequency of α in the population from which it is derived. For population I, allele frequencies are derived across clones in samples $1, \ldots, n$ under a multinomial-Dirichlet model (with a uniform prior over the standard simplex); for population C, we instead consider allele frequencies across clones in sample b. We make the simplifying assumptions of marker-wise independence (i.e., we assume that loci are unlinked); neutral markers; and clone-wise independence, both within samples and between non-recrudescent clones across samples (i.e., we ignore relatedness, which amplifies allelic sharing between closely-related clones [1]).

The principal difficulty arises because per-sample genotyping data are generated in bulk. We observe the presence or absence of detection of an allele within a sample, but not the allocation of alleles to individual clones (phasing), and thus not the per-sample allele counts. Here, we take a uniform distribution over all allelic configurations that are compatible with the set of observed alleles. Further complications include imperfect detectability of clones and inaccurate genotyping. Accordingly, we account for undetected clones in the paired baseline b and recurrent r samples, under a marker-wise truncated binomial model. The locus-wise probability of observations for sample r is formulated over successfully genotyped clones only. In contrast, allelic states for undetected clones in sample b are imputed based on population I allele frequencies (this imputation is the rationale for excluding sample b from population I). We also allow for genotyping error in baseline samples b and $1, \ldots, n$ relative to the recurrent sample r, specified by the probability that an allele called in a baseline sample matches an allele called in sample r.

In the following sections, we derive an analytic locus-wise likelihood of observed genotypes for sample r that addresses these complications systematically. We first formulate the locus-wise probability of observations for the recurrent sample r with respect to allele frequencies in populations I and C (Supplementary Note 1.1), followed by per-sample allele counts across clones in baseline samples b and $1, \ldots, n$ (Supplementary Note 1.2). Characterising per-sample allele counts from categorical (presence/absence) data on multiclonal samples is a non-trivial problem that has been addressed previously (see [2–6], which are all reviewed in [7]). However, to evaluate the likelihood, we note that it is sufficient to compute moments of allele counts summed over one or more baseline samples. This is a fundamentally easier problem, and permits an explicit solution under our simplifying assumptions (Supplementary Note 1.3).

Notation

We begin by introducing notation and several additional assumptions. We consider samples that have been genotyped at a panel of L markers. At locus $\ell \in \{1, \ldots, L\}$, for each sample z, we observe a set of alleles $G_\ell^{(z)}$. We denote by $M_\ell^{(z)} := |G_\ell^{(z)}|$ the observed cardinality (i.e., the number of distinct alleles) at locus ℓ for sample z. The MOI for sample z is taken to be the maximum observed cardinality across the set of L loci, that is,

$$M^{(z)} = \max_{1 \le \ell \le L} \left|G_\ell^{(z)}\right| \tag{1}$$

(this assumption can be readily relaxed).

We thus assume that sample z comprises a composite of $M^{(z)}$ clones. We index these clones j (arbitrary ordering), and denote by $\alpha_\ell^{(z,j)}$ the allele carried by the jth clone in the zth sample at locus ℓ. If $M^{(z)} > 1$ we do not observe $\alpha_\ell^{(z,j)}$. Assuming perfect detectability across clones and perfect genotyping accuracy, we can at most observe whether or not at least one clone in sample z carries allele α at locus ℓ, that is,

$$\mathbb{1}\{\alpha \in G_\ell^{(z)}\} = 1 - \prod_{j=1}^{M^{(z)}} \mathbb{1}\{\alpha_\ell^{(z,j)} \neq \alpha\}.$$

However, we do not observe how many or which (under the arbitrary ordering) of the $M^{(z)}$ clones within sample z carry allele α at locus ℓ.

We model genotyping error for baseline samples as follows. Denote by $H_\ell \supset G_\ell^{(r)} \bigcup G_\ell^{(b)} \bigcup_{i=1}^{n} G_\ell^{(i)}$ the set of known alleles at locus ℓ. We say that an allele identified as $\alpha \in H_\ell$ in a baseline sample b or $1, \ldots, n$ matches allele $\alpha' \in H_\ell$ in sample r with probability $\delta_\ell(\alpha, \alpha') \ge 0$ where

$$\sum_{\alpha' \in H_\ell} \delta_\ell(\alpha, \alpha') = 1 \text{ for each } \alpha \in H_\ell.$$

For notational convenience, we impose an arbitrary ordering on elements in the set H_ℓ; the notation $\boldsymbol{\theta}_\ell$ is used to denote a vector of allele frequencies, while $\mathbf{C}_\ell$ is used to denote a vector of per-sample allele counts across the ordered set H_ℓ. δ_ℓ can then be formulated as a right stochastic matrix (i.e.,with rows summing to 1). A summary of notation is provided in Table 1.

1.1 A locus-wise model predicated on allele frequencies

We begin by formulating a locus-wise model of observed alleles $G_\ell^{(r)}$ for sample r, with respect to allele frequencies in populations I and C. The derivation of allele frequencies is deferred to Supplementary Note 1.2.

To accommodate incomplete detection of clones in the recurrent sample r, we allocate observed alleles to successfully genotyped clones only. We adopt a locus-wise truncated multinomial model for the number of successfully genotyped clones at each locus ℓ. We assume that each clone in sample r, whether recrudescent or newly-inoculated, is successfully genotyped at locus ℓ with equal probability ω. Suppose

	Quantity	Interpretation
Observed	H_ℓ	Set of possible alleles at locus ℓ
	$G_\ell^{(z)}$	Set of alleles observed for sample z at locus ℓ
	$M_\ell^{(z)} = \lvert G_\ell^{(z)} \rvert$	Cardinality at locus ℓ for sample z
	$M^{(z)}$	Multiplicity of infection for sample z
User-specified	ω	Per-clone locus-wise probability of being detected at locus ℓ (applied to clones in samples b and r only)
	$\delta_\ell(\alpha, \alpha')$	Model of genotyping error: probability that an allele identified as α in a baseline sample b or $i = 1, \ldots, n$ matches allele α' in a recurrent sample r locus ℓ
Estimated	M_S	Number of clones in sample r derived from population $S \in \{I, C\}$
Unobserved	$\alpha_\ell^{(z,j)}$	True allele harboured by clone j of sample z at locus ℓ
Modelled	$\theta_\ell^{(P)}(\alpha)$	Frequency of allele α at locus ℓ in population $P \in \{I, C\}$
	$Q_\ell^{(r,S)}$	Number of clones in sample r that are derived from population $S \in \{I, C\}$ and have been detected at locus ℓ
	$Q_\ell^{(b)}$	Number of clones in sample b that have been detected at locus ℓ
	$C_\ell^{(i)}(\alpha)$	Count of allele α across clones in sample $i = 1, \ldots, n$ at locus ℓ
	$C_\ell^{(b,q)}(\alpha)$	Count of allele α across the q clones in sample b that have been genotyped (in)correctly at locus ℓ
	$X_\ell^{(z)}(A)$	Adjusting for genotyping error relative to sample r, the number of alleles observed for baseline sample $z = b$ or $z = 1, \ldots, n$ at locus ℓ that lie in the set $A \subset H_\ell$
Other	$s(m, k)$	Unsigned Stirling number of the first kind
	$S(m, k)$	Stirling number of the second kind
	$\mathcal{P}(X)$	Power set of X, i.e., the set of all subsets of X encompassing the empty set

Supplementary Table 1: Summary of notation

the recurrent sample r comprises $M_C = m$ clones from population C and $M_I = M^{(r)} - m$ clones from population I. Then the numbers of clones $Q_\ell^{(r,S)}$ in sample r that are derived from populations $S \in \{C, I\}$ and successfully genotyped at locus ℓ (that is, contribute to the observation $G_\ell^{(r)}$) take the form

$$\begin{aligned}
&\mathbb{P}\left(Q_\ell^{(r,C)} = q_C, Q_\ell^{(r,I)} = q_I, \mid M_C = m, M_I = M^{(r)} - m\right) \\
&\quad = \frac{\binom{m}{q_C}\binom{M^{(r)}-m}{q_I}\omega^{q_I+q_C}(1-\omega)^{M^{(r)}-q_I-q_C}}{\sum_{j=M_\ell^{(r)}}^{M^{(r)}}\binom{M^{(r)}}{j}\omega^j(1-\omega)^{M^{(r)}-j}} \text{ for } q_I + q_C \geq M_\ell^{(r)},\ q_C \leq m,\ q_I \leq M^{(r)} - m. \qquad (2)
\end{aligned}$$

Suppose $Q_\ell^{(r,C)} = q_C$ clones from population C and $Q_\ell^{(r,I)} = q_I$ clones from population I are successfully genotyped at locus ℓ in sample r. As in [5, 8], we use the inclusion-exclusion principle to average over the different ways to allocate alleles in $G_\ell^{(r)}$ to $q_C + q_I$ clones; the inclusion-exclusion principle circumvents the need to allocate alleles to clones explicitly. Let $\mathcal{P}(G_\ell^{(r)})$ denote the power set of $G_\ell^{(r)}$, that is, the set of all subsets of $G_\ell^{(r)}$ (encompassing the empty set). Then the conditional probability of the observation $G_\ell^{(r)}$ at locus ℓ is

$$\begin{aligned}
&\mathbb{P}\left(G_\ell^{(r)} \,\middle|\, \boldsymbol{\theta}_\ell^{(C)},\, \boldsymbol{\theta}_\ell^{(I)},\, Q_\ell^{(r,C)} = q_C,\, Q_\ell^{(r,I)} = q_I\right) \\
&\quad = \sum_{A \in \mathcal{P}\left(G_\ell^{(r)}\right)} (-1)^{|A|} \left(\sum_{\alpha \in G_\ell^{(r)} \setminus A} \theta_\ell^{(C)}(\alpha)\right)^{q_C} \left(\sum_{\alpha \in G_\ell^{(r)} \setminus A} \theta_\ell^{(I)}(\alpha)\right)^{q_I}, \qquad (3)
\end{aligned}$$

where $\theta_\ell^{(S)}(\alpha)$ is the frequency of allele α at locus ℓ in population $S \in \{I, C\}$.

Convolving Equation (3) over the distribution of successfully genotyped clones (2) then yields

$$\begin{aligned}
&\mathbb{P}\left(G_\ell^{(r)} \,\middle|\, \boldsymbol{\theta}_\ell^{(C)},\, \boldsymbol{\theta}_\ell^{(I)},\, M_C = m,\ M_I = M^{(r)} - m\right) \\
&\quad = \sum_{q_C=\max\{0, M_\ell^{(r)} - (M^{(r)}-m)\}}^{m} \ \sum_{q_I = \max\{0, M_\ell^{(r)} - q_C\}}^{M^{(r)}-m} \frac{\binom{m}{q_C}\binom{M^{(r)}-m}{q_I}\omega^{q_I+q_C}(1-\omega)^{M^{(r)}-q_I-q_C}}{\sum_{j=M_\ell^{(b)}}^{M^{(r)}}\binom{M^{(r)}}{j}\omega^j(1-\omega)^{M^{(r)}-j}} \\
&\qquad \left\{\sum_{A \in \mathcal{P}\left(G_\ell^{(r)}\right)} (-1)^{|A|} \left(\sum_{\alpha \in G_\ell^{(r)} \setminus A} \theta_\ell^{(C)}(\alpha)\right)^{q_C} \left(\sum_{\alpha \in G_\ell^{(r)} \setminus A} \theta_\ell^{(I)}(\alpha)\right)^{q_I}\right\}. \qquad (4)
\end{aligned}$$

Remark: Rather than modelling (un)genotyped clones in recurrent sample r directly, we could have taken a uniform distribution over all allelic configurations of $M^{(r)}$ clones such that each allele in the observed set $G_\ell^{(r)}$ is carried by at least one clone at locus ℓ; this would implicitly allow for the incomplete detection of clones in sample r. Under this construction, the conditional probability of the observation $G_\ell^{(r)}$ at locus ℓ would take the form

$$\begin{aligned}
&\mathbb{P}_{\text{other}}\left(G_\ell^{(r)} \,\middle|\, \boldsymbol{\theta}_\ell^{(C)},\, \boldsymbol{\theta}_\ell^{(I)},\, M_C = m,\ M_I = M^{(r)} - m\right) \\
&\quad = \sum_{A \in \mathcal{P}\left(G_\ell^{(r)}\right)} (-1)^{|A|} \left(\sum_{\alpha \in H_\ell \setminus A} \theta_\ell^{(C)}(\alpha)\right)^{m} \left(\sum_{\alpha \in H_\ell \setminus A} \theta_\ell^{(I)}(\alpha)\right)^{M^{(r)}-m} \qquad (5)
\end{aligned}$$

with differences to Equation (3) highlighted in red. We have not adopted this construction because it is liable to generate false positive signals of recrudescence when the recurrent sample r has MOI>1, and exhibits alleles matching the paired baseline sample b at a subset of loci.

1.2 Modelling allele frequencies

Here, we address the derivation of allele frequencies for both populations I and C. By convolving Equation (4) over the modelled distribution of population allele frequencies, we show that the locus-wise probability of observations $G_\ell^{(r)}$ for sample r can be formulated as a multinomial expression of pooled-sample allele counts (i.e, allele counts summed over one or more baseline samples b and $i = 1, \ldots, n$). The consequences of genotyping error for baseline samples relative to the recurrent sample r, specified by the probability that alleles called in baseline samples b and $i = 1, \ldots, n$ match alleles called in the recurrent sample r, will be addressed in Supplementary Note 1.3 in the sub-model of per-sample allele counts.

1.2.1 Allele frequencies for population I

Treating samples $1, \ldots, n$ as a finite sample from a larger population I, we derive allele frequencies for population I under a Bayesian multinomial-Dirichlet model. We weight each sample by its MOI, whereby each clone in samples $i = 1, \ldots, n$ is weighted equally.

For notational convenience, we introduce an arbitrary ordering on H_ℓ, the set of possible alleles at locus ℓ. For the kth allele α_k in the set H_ℓ, we denote by $\theta_\ell^{(I)}(k)$ the baseline population frequency, and by

$$C_\ell^{(i)}(k) = \sum_{j=1}^{M^{(i)}} \mathbb{1}\{\alpha_\ell^{(i,j)} = \alpha_k\} \tag{6}$$

the per-sample allele count $\in \{0, 1, \ldots, M^{(i)}\}$ across clones in sample $1, \ldots, n$.

Under the assumed independence of clones within and between the baseline samples $1, \ldots, n$, we model pooled-sample allele counts aggregated over samples $1, \ldots, n$ to follow a multinomial distribution

$$\sum_{i=1}^{n} \mathbf{C}_\ell^{(i)} \,\Big|\, \boldsymbol{\theta}_\ell^{(I)} \sim \text{Multinomial}\left(\sum_{i=1}^{n} M^{(i)},\, \boldsymbol{\theta}_\ell^{(I)}\right)$$

governed by the population allele frequencies $\boldsymbol{\theta}_\ell^{(I)}$.

Taking a uniform prior for $\boldsymbol{\theta}_\ell^{(I)}$ over the $|H_\ell| - 1$ simplex, $\text{Dirichlet}\big(|H_\ell|,\, \mathbf{1}\big)$, the posterior distribution of allele frequencies $\boldsymbol{\theta}_\ell^{(I)}$ given the per-sample allele counts $\mathbf{C}_\ell^{(i)}$ takes the form

$$\boldsymbol{\theta}_\ell^{(I)} \,\Big|\, \sum_{i=1}^{n} \mathbf{C}_\ell^{(i)} \sim \text{Dirichlet}\left(\sum_{i=1}^{n} \mathbf{C}_\ell^{(i)} + \mathbf{1}\right). \tag{7}$$

1.2.2 Allele frequencies for population C

Allele frequencies for population C are largely governed by clones in the paired baseline sample b. However, we accommodate undetected clones in sample b, with allelic states for ungenotyped clones imputed using population I allele frequencies $\boldsymbol{\theta}_\ell^{(I)}$. This imputation is the rationale for withholding the paired sample b when deriving population I allele frequencies.

Akin to the model of (un)genotyped clones for sample r (Equation (2)), we model each clone in sample b to be detected at locus ℓ with probability ω. Then the number of clones $Q_\ell^{(b)}$ in sample b that are successfully genotyped at locus ℓ (that is, contribute to the observation $G_\ell^{(b)}$) is modelled to follow a truncated binomial distribution, with the lower bound of the support given by the observed cardinality $M_\ell^{(b)}$ at locus ℓ:

$$\mathbb{P}\left(Q_\ell = q \,|\, M_\ell^{(b)}, M^{(b)}, \omega\right) = \frac{\binom{M^{(b)}}{q}\omega^q (1-\omega)^{M^{(b)}-q}}{\sum_{j=M_\ell^{(b)}}^{M^{(b)}} \binom{M^{(b)}}{j}\omega^j (1-\omega)^{M^{(b)}-j}} \text{ for } q = M_\ell^{(b)}, \ldots, M^{(b)}. \tag{8}$$

Given $Q_\ell^{(b)} = q$, let $\mathbf{C}_\ell^{(b,q)}$ be the vector of allele counts across the q clones in sample b that have been successfully genotyped at locus ℓ. Then, conditional on $Q_\ell^{(b)} = q$, $\mathbf{C}_\ell^{(b,q)}$ and $\boldsymbol{\theta}_\ell^{(I)}$, we model

$$\boldsymbol{\theta}_\ell^{(C)} \,\middle|\, \left\{ Q_\ell^{(b)} = q,\, \mathbf{C}_\ell^{(b,q)},\, \boldsymbol{\theta}_\ell^{(I)} \right\} = \frac{\mathbf{C}_\ell^{(b,q)}}{M^{(b)}} + \left(1 - \frac{q}{M^{(b)}}\right) \cdot \boldsymbol{\theta}_\ell^{(I)}. \tag{9}$$

Remark: Assuming perfect detection of clones in the paired baseline sample b (i.e., setting $\omega = 1$) can have strong implications when paired recurrences share alleles at all but one locus, with a length difference that cannot plausibly be explained by genotyping error, but likely reflects human error; see Figure 1 for three empirical examples from [9]. Accommodating ungenotyped clones in sample b can prevent a single locus from having a disproportionately large effect on overall classifications.

1.2.3 A locus-wise model predicated on allele counts

Using the derived distributions of allele frequencies for population I, $\boldsymbol{\theta}_\ell^{(I)}$ (Equation (7)), and population C, $\boldsymbol{\theta}_\ell^{(C)}$ (Equation (9)), we can formulate the locus-wise probability of observations $G_\ell^{(r)}$ for sample r in terms of sample allele counts.

Substituting Equation (9) into Equation (4) and convolving over the truncated binomial distribution $Q_\ell^{(b)}$ (8) governing the number of clones detected at locus ℓ in sample b, we find that:

$$\begin{aligned}
&\mathbb{P}\left(G_\ell^{(r)} \,\middle|\, \hat{\boldsymbol{\theta}}_\ell^{(I)},\, \mathbf{C}_\ell^{(b,q)},\, M_C = m,\, M_I = M^{(r)} - m\right) \\
&= \frac{1}{J_\ell^{(b)} J_\ell^{(r)}} \sum_{q_B = M_\ell^{(b)}}^{M^{(b)}} \sum_{q_C = \max\{0, M_\ell^{(r)} - (M^{(r)} - m)\}}^{m} \sum_{q_I = \max\{0, M_\ell^{(r)} - q_C\}}^{M^{(r)} - m} \binom{M^{(b)}}{q_B} \binom{m}{q_C} \binom{M^{(r)} - m}{q_I} \omega^{q_I + q_C + q_B} (1-\omega)^{M^{(r)} + M^{(b)} - q_I - q_C - q_B} \\
&\quad \sum_{k=0}^{q_C} \binom{q_C}{k} \left(\frac{q_B}{M^{(b)}}\right)^k \left(1 - \frac{q_B}{M^{(b)}}\right)^{q_C - k} \sum_{A \in \mathcal{P}\left(G_\ell^{(r)}\right)} (-1)^{|A|} \left(\sum_{\alpha \in G_\ell^{(r)} \setminus A} \frac{C_\ell^{(b,q_B)}(\alpha)}{q_B}\right)^k \left(\sum_{\alpha \in G_\ell^{(r)} \setminus A} \theta_\ell^{(I)}(\alpha)\right)^{q_I + q_C - k}
\end{aligned} \tag{10}$$

where we set

$$J_\ell^{(s)} = \sum_{j = M_\ell^{(s)}}^{M^{(s)}} \binom{M^{(s)}}{j} \omega^j (1-\omega)^{M^{(s)} - j}. \tag{11}$$

Convolving Equation (10) over the derived distribution of $\boldsymbol{\theta}_\ell^{(I)}$ (7) (with integration performed over the $|H_\ell| - 1$ simplex) yields the locus-wise probability of observations $G_\ell^{(r)}$ for sample r formulated with respect to the per-sample allele counts $\mathbf{C}_\ell^{(b,q)}$ across successfully genotyped clones in sample b, and the pooled-sample allele counts $\sum_{i=1}^n \mathbf{C}_\ell^{(i)}$:

$$\mathbb{P}\left(G_\ell^{(r)} \,\middle|\, \sum_{i=1}^{n} \mathbf{C}_\ell^{(i)},\, \mathbf{C}_\ell^{(b,q)},\, M_C = m,\; M_I = M^{(r)} - m\right)$$

$$= \int_{\boldsymbol{\theta}_\ell} \mathbb{P}\left(G_\ell^{(r)} \,\middle|\, \boldsymbol{\theta}_\ell^{(I)},\, C_\ell^{(b,q)},\, M_C = m,\; M_I = M^{(r)} - m\right) \cdot f\left(\boldsymbol{\theta}_\ell \,\middle|\, \sum_{i=1}^{n} \mathbf{C}_\ell^{(i)}\right) d\boldsymbol{\theta}_\ell$$

$$= \frac{1}{J_\ell^{(b)} J_\ell^{(r)}} \sum_{q_B = M_\ell^{(b)}}^{M^{(b)}} \sum_{q_C = \max\{0, M_\ell^{(r)} - (M^{(r)} - m)\}}^{m} \sum_{q_I = \max\{0, M_\ell^{(r)} - q_C\}}^{M^{(r)} - m} \binom{M^{(b)}}{q_B} \binom{m}{q_C} \binom{M^{(r)} - m}{q_I} \omega^{q_I + q_C + q_B} (1 - \omega)^{M^{(r)} + M^{(b)} - q_I - q_C - q_B}$$

$$\sum_{k=0}^{q_C} \binom{q_C}{k} \left(\frac{q_B}{M^{(b)}}\right)^k \left(1 - \frac{q_B}{M^{(b)}}\right)^{q_C - k} \frac{\Gamma\left(\sum_{i=1}^{n} M^{(i)} + |H_\ell|\right)}{\Gamma\left(\sum_{i=1}^{n} M^{(i)} + |H_\ell| + q_I + q_C - k\right)}$$

$$\left\{ \sum_{A \in \mathcal{P}\left(G_\ell^{(r)}\right)} (-1)^{|A|} \left(\sum_{\alpha \in G_\ell^{(r)} \setminus A} \frac{C_\ell^{(b, q_B)}(\alpha)}{q_B} \right)^k \left(|G_\ell^{(r)}| - |A| + \sum_{\alpha \in G_\ell^{(r)} \setminus A} \sum_{i=1}^{n} C_\ell^{(i)}(\alpha) \right)^{\left(q_I + q_C - k\right)} \right\} \tag{12}$$

where $(\cdot)^{(\cdot)}$ denotes the rising factorial. Here, we have used the aggregation property of the Dirichlet distribution.

To write Equation (12) in terms of simple powers of $\mathbf{C}_\ell^{(b,q)}$ and $\sum_{i=1}^n \mathbf{C}_\ell^{(i)}$, we apply the binomial expansion to yield

$$\left(|G_\ell^{(r)}| - |A| + \sum_{\alpha \in G_\ell^{(r)} \setminus A} \sum_{i=1}^{n} C_\ell^{(i)}(\alpha) \right)^{\left(q_I + q_C - k\right)}$$

$$= \sum_{j=0}^{q_I + q_C - k} \beta_{(q_I + q_C - k,\, j)}\left(|G_\ell^{(r)}| - |A|\right) \cdot \left(\sum_{\alpha \in G_\ell^{(r)} \setminus A} \sum_{i=1}^{n} C_\ell^{(i)}(\alpha) \right)^j \Bigg\} \tag{13}$$

where the coefficients

$$\beta_{(m,j)}(x) = \sum_{k=j}^{m} s(m,k) \binom{k}{j} x^{k-j} \tag{14}$$

are formulated with respect to unsigned Stirling numbers of the first kind $s(m,k)$. Substituting Equation (13) into Equation (12) yields a multinomial expression of the per-sample allele counts $\mathbf{C}^{(b,q)}$ and the pooled-sample allele counts $\sum_{i=1}^n \mathbf{C}_\ell^{(i)}$, as desired.

1.3 Moments of allele counts

Using bulk genotypic data, allele counts are not directly observed for multiclonal samples. To recover the locus-wise likelihood of observations $G_\ell^{(r)}$ for sample r given it comprises $M_C = m$ recrudescent and $M_I = M^{(r)} - m$ newly-inoculated clones, we must convolve Equation (12) over the underlying sample-wise distribution of allele counts. Because the locus-wise probability of observations $G_\ell^{(r)}$ for sample r can be written as a multinomial expression of the per-sample allele counts $\mathbf{C}^{(b,q)}$ and pooled-sample allele

counts $\sum_{i=1}^{n} \mathbf{C}_\ell^{(i)}$ (Equations (12) and (13)), evaluation of the locus-wise likelihood distils down to the computation of moments of these allele counts. This is a tractable combinatorial problem which can be solved explicitly under our simplifying assumptions.

Substituting Equation (13) into Equation (12) and convolving over the distribution of per-sample allele counts for baseline samples b and $i = 1, \ldots, n$, we obtain

$$
\begin{aligned}
&\mathcal{L}\Big(G_\ell^{(r)} \Big| M_C = m,\ M_I = M^{(r)} - m\Big) \\
&= \sum_{\mathbf{C}_\ell^{(b,q)}} \sum_{\mathbf{C}_\ell^{(i)}} \mathbb{P}\left(G_\ell^{(r)} \middle| \sum_{i=1}^{n} \mathbf{C}_\ell^{(i)},\ \mathbf{C}_\ell^{(b,q)}, M_C = m,\ M_I = M^{(r)} - m\right) \mathbb{P}\left(\sum_{i=1}^{n} \mathbf{C}_\ell^{(i)}\right) \mathbb{P}\left(\mathbf{C}_\ell^{(b,q)}\right) \\
&= \frac{1}{J_\ell^{(b)} J_\ell^{(r)}} \sum_{q_B = M_\ell^{(b)}}^{M^{(b)}} \sum_{q_C = \max\{0, M_\ell^{(r)} - (M^{(r)} - m)\}}^{m} \sum_{q_I = \max\{0, M_\ell^{(r)} - q_C\}}^{M^{(r)} - m} \binom{M^{(b)}}{q_B} \binom{m}{q_C} \binom{M^{(r)} - m}{q_I} \omega^{q_I + q_C + q_B} (1 - \omega)^{M^{(r)} + M^{(b)} - q_I - q_C - q_B} \\
&\quad \sum_{k=0}^{q_C} \binom{q_C}{k} \left(\frac{q_B}{M^{(b)}}\right)^k \left(1 - \frac{q_B}{M^{(b)}}\right)^{q_C - k} \frac{\Gamma\big(\sum_{i=1}^{n} M^{(i)} + |H_\ell|\big)}{\Gamma\big(\sum_{i=1}^{n} M^{(i)} + |H_\ell| + q_I + q_C - k\big)} \\
&\qquad \Bigg\{ \sum_{A \in \mathcal{P}\left(G_\ell^{(r)}\right)} (-1)^{|A|}\, \mathbb{E}\left[\left(\sum_{\alpha \in G_\ell^{(r)} \setminus A} \frac{C_\ell^{(b,q_B)}(\alpha)}{q_B}\right)^k\right] \\
&\qquad\qquad \sum_{j=0}^{q_I + q_C - k} \beta_{(q_I + q_C - k,\, j)} \big(|G_\ell^{(r)}| - |A|\big)\, \mathbb{E}\left[\left(\sum_{\alpha \in G_\ell^{(r)} \setminus A} \sum_{i=1}^{n} C_\ell^{(i)}(\alpha)\right)^j\right] \Bigg\}
\end{aligned}
\tag{15}
$$

where we have evoked the assumed independence of (genotyped) clones across baseline samples b and $1, \ldots, n$.

Evaluating the locus-wise likelihood of observations $G_\ell^{(r)}$ for sample r (15) necessitates the calculation of moments of the collective counts of allelic subsets A' summed over one or more baseline samples. We can derive these moments explicitly under three key assumptions:

1. We consider the distribution of per-sample allele counts $C_\ell^{(i)}$ for each sample $i = 1, \ldots, n$, such that all of the $M^{(i)}$ clones in sample i contribute to the observation $G_\ell^{(i)}$ at locus ℓ, whereby we assume implicitly that the effects of ungenotyped clones (if any) 'average out' across samples $i = 1, \ldots, n$ when formulating allele frequencies for population I; note that $C_\ell^{(b,q)}$ corresponds explicitly to the $Q_\ell = q$ clones within sample b that have been successfully genotyped at locus ℓ and therefore contribute to the observation $G_\ell^{(b)}$.

2. We model clones to be independent within and between baseline samples.

3. We take a uniform distribution over compatible allelic configurations within each sample, that is, any allocation of the alleles $G_\ell^{(i)}$ ($G_\ell^{(b)}$) over $M^{(i)}$ ($q = M_\ell^{(b)}, \ldots, M^{(b)}$) clones, such that at least one clone harbours each allele in the set $G_\ell^{(i)}$ ($G_\ell^{(b)}$) and no clones harbour alleles outside the set $G_\ell^{(i)}$ ($G_\ell^{(b)}$), is modelled to be equally likely.

As foreshadowed in Supplementary Note 1.2, we adjust allele counts in baseline samples for genotyping error relative to the recurrent sample r, in accordance with the non-parametric model $\delta_\ell(\alpha, \alpha')$ which

yields the probability that an allele identified as $\alpha \in H_\ell$ in a baseline sample matches allele $\alpha' \in H_\ell$ in a recurrent sample r.

1.3.1 Per-sample allele counts

We begin by characterising per-sample allele counts $\mathbf{C}_\ell^{(b,q)}$ for the q clones in sample b that are successfully genotyped at locus ℓ. Denote by

$$X_\ell^{(b)}(A') = \left| \left\{ \bigcup_{j=1}^{q} \alpha_\ell^{(b,j)} \right\} \cap A' \right|$$

the number of distinct alleles, harboured by the q clones in sample b that are successfully genotyped at locus ℓ, that lie in a specified set $A' \subset H_\ell$. Accommodating genotyping error relative to the recurrent sample r, each observed allele $g \in G_\ell^{(b)}$ matches an allele within the set $A' \subset G_\ell^{(r)}$ with probability

$$\delta(g, A') = \sum_{\alpha \in A'} \delta_\ell(g, \alpha).$$

We model genotyping error independently for each allele in the observed set $G_\ell^{(b)}$. Accordingly, we model $X_\ell^{(b)}(A')$ as a sum of $M_\ell^{(b)}$ independent but not identically distributed Bernoulli random variables (i.e., a Poisson binomial distribution) with respective success probabilities $\delta(g, A')$, $g \in G_\ell^{(b)}$.

Define

$$C_\ell^{(b,q)}(A') = \sum_{\alpha \in A'} C_\ell^{(b,q)}(\alpha) = \sum_{j=1}^{q} \mathbb{1}\{\alpha_\ell^{(b,j)} \in A'\} \tag{16}$$

to be the number of clones within sample b that are genotyped (correctly or incorrectly) at locus ℓ and harbour an allele in the set A' at locus ℓ. We can recover the distribution of $C_\ell^{(b,q)}(A')$ using a simple combinatorial argument as follows.

The number of allelic configurations $N_\ell^{(b,q)}$ of q clones compatible with the observation $G_\ell^{(b)}$ at locus ℓ is equivalent to the number of ordered partitions of q objects into $M_\ell^{(b)}$ non-empty subsets. We can write this in the form

$$N_\ell^{(b,q)} = S\big(q, M_\ell^{(b)}\big) \cdot M_\ell^{(b)}!$$

where $S(n, k)$ denotes the Stirling number of the second kind.

Given $X_\ell^{(b)}(A') = h$, allelic configurations of q clones such that $C_\ell^{(q,b)}(A') = k \geq h$ clones harbour alleles in the set A' can be generated by:

- Separating the q clones into two groups of size k and $q - k$ respectively, which can be performed in $\binom{q}{k}$ ways;
- For the group of size k (corresponding to clones with alleles in the set A'), constructing ordered partitions into h non-empty subsets, which can be performed in $S(k, h) \cdot h!$ ways;

- For the group of size $(q-k)$ (corresponding to clones with alleles outside the set A'), constructing ordered partitions into $\big(M_\ell^{(b)}-h\big)$ non-empty subsets, which can be performed in $S\big(q-k, M_\ell^{(b)}-h\big)\cdot\big(M_\ell^{(b)}-h\big)!$ ways.

Therefore, given $X_\ell^{(b)}(A')=h$, the number of allelic configurations of q clones, such that the unique set of observed alleles at locus ℓ is $G_\ell^{(b)}$ and precisely $k\geq h$ clones harbour alleles within the set A', takes the form

$$N_\ell^{(b,q)}(A',k,h)=\binom{q}{k}\cdot S(k,h)h!\cdot S\big(q-k,M_\ell^{(b)}-h\big)\big(M_\ell^{(b)}-h\big)!$$

We thus obtain the probability mass function for the number of clones $C_\ell^{(b,q)}(A')$ (of the q clones that are genotyped at locus ℓ in sample b) with alleles lying in the set A' at locus ℓ:

$$\mathbb{P}\big(C_\ell^{(b,q)}(A')=k\big)=\sum_{h=\max\{0,M_\ell^{(b)}-q+k\}}^{\min\{k,M_\ell^{(b)}\}}\frac{\binom{q}{k}}{\binom{M_\ell^{(b)}}{h}}\cdot\frac{S(k,h)S\big(q-k,M_\ell^{(b)}-h\big)}{S(q,M_\ell^{(b)})}\cdot\mathbb{P}(X_\ell^{(b)}(A')=h)\qquad(17)$$

for $k\in\{0,1,\ldots,q\}$.

We can use the PMF to (17) to compute moments of $C^{(b,q)}(A')$ for relevant allelic subsets A' to be substituted into Equation (15). Efficient computation of the probability mass function $\mathbb{P}(X_\ell^{(b)}(A')=h)$ of the Poisson binomial distribution can be performed using the DFT method of Hong [10], with an implementation available in the R package `poisbinom` [11]. The consequences of genotyping error, relative to the recurrent sample r, are embedded implicitly within the term $\mathbb{P}\big(X_\ell^{(b)}(A')=h\big)$.

1.3.2 Pooled-sample allele counts

The likelihood (15) additionally requires computation of the moments of sample counts of allelic sets A':

$$C_\ell^{(I)}(A'):=\sum_{i=1}^{n}\sum_{\alpha\in A'}C_\ell^{(i)}(\alpha).$$

Here, we exploit the fact that the order of these moments (up to $M^{(r)}$) is likely to be smaller than the size of the baseline sample n. With minor modifications in notation, we can use the PMF (17) for per-sample allele counts to compute the yth cumulant $\kappa_y\big(C_\ell^{(i)}(A')\big)$ directly for the number of clones $C_\ell^{(i)}(A')=\sum_{\alpha\in A'}C_\ell^{(i)}(\alpha)$ within each sample $i=1,\ldots,n$ that harbour alleles within the set A'. As clones across baseline samples are independent, it follows that

$$\kappa_y\big(C_\ell^{(I)}(A')\big)=\sum_{i=1}^{n}\kappa_y\big(C_\ell^{(i)}(A')\big)\qquad(18)$$

To recover the mth moment of $C_\ell^{(I)}(A')$, we exploit the mapping between moments and cumulants:

$$\mathbb{E}\left[C_\ell^{(I)}(A')^m\right]=B_m\Big(\kappa_1\big(C_\ell^{(I)}(A')\big),\ldots,\kappa_m\big(C_\ell^{(I)}(A')\big)\Big).\qquad(19)$$

where $B_m(\cdot)$ denotes the mth complete exponential Bell polynomial. Equation (19) can then be substituted into the likelihood (15) for relevant allelic subsets A'.

2 Validation against simulated data

To validate our classification model PfRecur, we simulate recurrent samples as mixtures of newly-inoculated and recrudescent clones. A summary of simulation parameters is provided in Table 2.

**Parameter**	**Interpretation**	**Value**
L	Number of independent (unlinked) and neutral markers	7
$\mathbf{h}$	Vector of marker cardinalities (number of possible alleles $\lvert H_\ell \rvert$ at each marker $\ell = 1, \ldots, L$)	$(30, 30, 20, 20, 20, 10)$
ε	Probability of genotyping error per allele call in each baseline sample	0.05
M_{max}	Maximum MOI (number of not necessarily distinct clones) within each sample	9
μ	Mean of the geometric distribution (with non-zero state space) governing the sample MOI (truncated at M_{max})	3
p_{sib}	Parameter governing within-sample sibling structure (represented by non-crossing partitions); zero corresponds to the absence of sibling structure, i.e., independent clones	0.1
ω	Per-clone marker-wise probability of detection	0.9
n	Number of baseline samples	25

Supplementary Table 2: Summary of simulation parameters

We generate baseline samples comprising n independent samples, accompanied by paired recurrent samples. For each sample, we simulate genotypic data across L markers, indexed $\ell = 1, \ldots, L$.

Each marker ℓ is uniquely specified by a cardinality h_ℓ. The set of possible alleles at marker ℓ is denoted $H_\ell = \{1, \ldots, h_\ell\}$, with the integer label interpreted as a length (in arbitrary units).

We accommodate structured genotyping error at each marker ℓ. We adopt a normalised geometric model, based on the absolute length difference between an observed and underlying allele [12], with success parameter $(1 - \varepsilon)$. We normalise this model such that each allele is genotyped erroneously with probability ε, that is,

$$\delta_\ell(i, j) = \begin{cases} \frac{\varepsilon^{|i-j|+1}}{\sum_{k \neq i} \varepsilon^{|i-k|}} & \text{if } j \neq i \\ 1 - \varepsilon & \text{if } j = i. \end{cases} \qquad (20)$$

We then generate a random permutation σ of the set H_ℓ. The population frequency of the $\sigma(i)^{\text{th}}$ allele at marker ℓ is taken to be

$$\theta_\ell\big(\sigma(i)\big) = \frac{2i}{h_\ell(h_\ell + 1)},$$

yielding the effective cardinality [13]

$$\text{EffectiveCardinality}_\ell = \left(\sum_{i=1}^{h_\ell} \theta_\ell(i)^2\right)^{-1} = \frac{3h_\ell}{4} + \frac{3}{8}\left(1 - \frac{1}{1+2h_\ell}\right).$$

This is motivated by empirical data, which appear to yield an approximately-linear trend in ordered (expected) sample allele frequencies.

The MOI of each sample (baseline or recurrent) is sampled from the geometric distribution with mean μ and non-zero state space, truncated at $M_{\max}$, that is,

$$P(M = m) = \frac{\mu^{M_{\max}-m}\big(\mu - 1\big)^{m-1}}{\mu^{M_{\max}} - \big(\mu - 1\big)^{M_{\max}}} \text{ for } m = 1, \ldots, M_{\max}. \tag{21}$$

We accommodate the presence of siblings within simulated samples. To generate a baseline set of clones for sample i with MOI $M^{(i)}$, we first simulate a non-crossing partition of the set $\{1, \ldots, M^{(i)}\}$, governed by the partition parameter p_{sib}: given

$$b_j \overset{i.i.d.}{\sim} \text{Bernoulli}(p_{\text{sib}}),\ j = 1, \ldots, (M^{(i)} - 1),$$

clones j and $(j+1)$ are assigned to the same block if and only if $b_j = 0$. For each block of siblings B, we then generate a pair of independent parents. The allelic state $\rho_\ell^{(q)}(B)$ of parent $q \in \{1, 2\}$ of block B at locus ℓ is sampled from a categorical distribution governed by the population allele frequencies

$$\rho_\ell^{(q)}(B) \sim \text{Categorical}(\boldsymbol{\theta}_\ell).$$

We then recover each clone in block B as a mosaic of these independent parents, retaining the assumption of unlinked markers: for each clone $j \in B$, the allelic state at marker ℓ

$$\alpha_\ell^{(i,j\in B)} \sim \text{Uniform}\big\{\rho_\ell^{(1)}(B), \rho_\ell^{(2)}(B)\big\}.$$

For each clone $j = 1, \ldots, M^{(i)}$, we simulate a Bernoulli random variable

$$\omega^{(i,j)} \overset{i.i.d.}{\sim} \text{Bernoulli}(\omega)$$

governing detection due to genotyping at locus ℓ. We then aggregate the set of detected alleles present at locus ℓ

$$G_\ell^{(\text{detected})} = \big\{\alpha^{(i,j)} \,\big|\, \omega^{(i,j)} = 1\big\}.$$

For each detected allele $g \in G_\ell^{(\text{detected})}$, we sample an observed allele from the categorical distribution

$$\gamma(g) \sim \text{Categorical}\big(\delta_\ell(g, 1), \ldots, \delta_\ell(g, h_\ell)\big);$$

classification and population allele frequency estimation is performed based on the observed set of genotypes $\gamma(G_\ell^{(\text{detected})})$ for the baseline sample.

For the ith baseline sample of MOI $M^{(i)}$, we generate a set of paired recurrences as follows. For each $M_{\text{recurrent}}^{(i)} = 1, \ldots, M_{\max}$ and $m = 0, \ldots, \min(M^{(i)}, M_{\text{recurrent}}^{(i)})$, we simulate a mixture of m recrudescent

and $(M^{(i)}_{\text{recurrent}} - m)$ newly-inoculated clones. The m recrudescent clones are sampled uniformly at random without replacement from the paired baseline sample; the $(M^{(i)}_{\text{recurrent}} - m)$ newly-inoculated clones are simulated under the same framework as a baseline sample. We account for the incomplete detection of clones in the recurrent sample (with the per-clone marker-wise probability of detection ω), but do not account for genotyping error for the recurrent sample.

Paired simulated recurrences are classified using our novel model PfRecur, with a uniform prior over the number of newly-inoculated vs recrudescent clones (i.e., with $\beta = 1$). Classification is based on the maximum observed cardinality across simulated markers, rather than the true MOI of each sample. We perform classification in an idealised setting with the user-specified parameters $\omega = 0.9$ and $\varepsilon = 0.05$ set to the values under which data were simulated.

Results in the main text are aggregated across 40 simulation runs (i.e., 40 simulated sets of baseline samples), amounting to 29077 simulated recurrent samples.

3 Empirical data: a re-analysis of Dimbu *et al.* [9]

3.1 Within-host diversity

To ascertain the contribution of each microsatellite marker to the MOI of a given sample, we derive a within-host diversity metric. For a sample with MOI $M^{(i)}$ and cardinality $M_\ell^{(i)}$ at marker ℓ, we define the within-host diversity of marker ℓ to be the expected value of the complement of Nei's gene identity metric (or equivalently, the probability of differing alleles at marker ℓ when a pair of clones is sampled with replacement from the sample), taking a uniform distribution over all possible allelic configurations of $M^{(i)}$ clones that harbour precisely $M_\ell^{(i)}$ distinct alleles at locus ℓ. Following the reasoning in Supplementary Note 1.3.1, this takes the form

$$\xi_\ell^{(i)} = 1 - \sum_{k=1}^{M^{(i)}-M_\ell^{(i)}+1} \binom{M^{(i)}}{k} \frac{S(k,1)S(M^{(i)}-k, M_\ell^{(i)}-1)}{S(M^{(i)}, M_\ell^{(i)})} \left(\frac{k}{M^{(i)}}\right)^2 \tag{22}$$

where $S(\cdot,\cdot)$ denotes a Stirling number of the second kind.

3.2 Sensitivity of PfRecur to user-specified parameters

Here, we consider the sensitivity of our classification model PfRecur to the user-specified parameters ε (governing the genotyping error probability under the normalised geometric model (20), applied to each allele called in a baseline sample relative to the recurrent sample of interest) and ω (governing the marker-wise probability of detection for each clone in the paired baseline and recurrent samples of interest). A complete sensitivity analysis is available in an accompanying GitHub repository (`https://github.com/somyamehra/PfTreatmentFailure`); below, we highlight samples for which classification is sensitive to user-specified parameters.

3.2.1 Classification of recurrences in Dimbu *et al.* [9]

The imperfect detection of clones in the paired baseline and recurrent sample proves a key consideration for a subset of recurrences in Dimbu *et al.* [9] using PfRecur. The posterior probability of at least one recrudescent clone (metric M1) is generally inflated as ungenotyped clones are increasingly accommodated (i.e., the per-clone marker-wise probability of detection ω is decreased, Supplementary Figure 1A). However, accommodating undetected clones in the paired baseline sample protects against plausible human error, such as the incorrect entry of a single allele in a putative recrudescence (Supplementary Figure 1B). Inferred mixtures of newly-inoculated vs recrudescent clones, quantified through the posterior proportion of recrudescent clones (metric M2), are also somewhat sensitive to per-clone marker-wise probability of detection ω in several cases (Supplementary Figure 1C). A dependence on the genotyping error probability ε, under the normalised geometric model (20), is also apparent for a subset of samples (Supplementary Figure 2); however, our results are largely insensitive to genotyping error probability within the plausible range $0.01 \leq \varepsilon \leq 0.1$. By default, we set $\varepsilon = 0.05$ and $\omega = 0.9$.

(A) Classification sensitive to per-clone marker-wise probability of detection

(A1)

	Baseline (LL21-061D0) Parasitemia: 5.62e+04/µL Temperature: 37.1	Recurrent (LL21-061D21) Parasitemia: 3.67e+03/µL Temperature: 36.6
M313	242, 248	242
M383	141	137
TA1	165, 168	174
POLYA	153	147
PFPK2	168, 171, 186	186
M2490	81	81
TA109	160, 172, 175	172

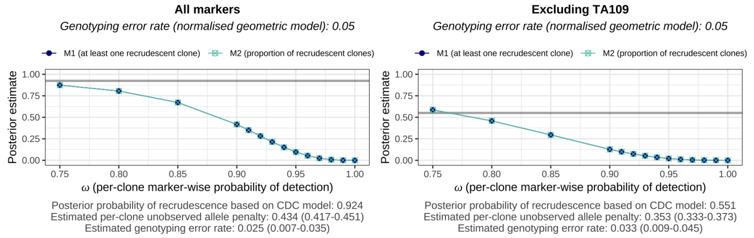

(A2)

	Baseline (ZL21-233D0) Parasitemia: 2.06e+03/µL Temperature: 37.5	Recurrent (ZL21-233D28) Parasitemia: 4.15e+03/µL Temperature: 36.4
M313	234	236
M383	147	147
TA1	177	168
POLYA	141, 144	144, 162
PFPK2	186	186
M2490	72	81
TA109	160, 175	163

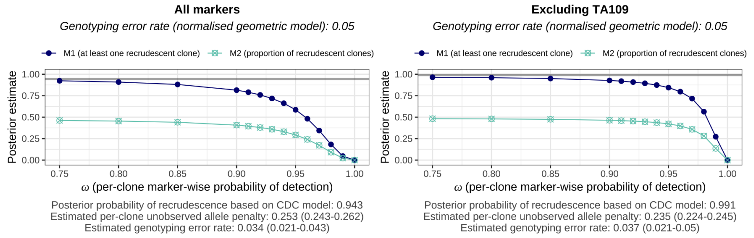

(A3)

	Baseline (ZQ21-085D0) Parasitemia: 5.01e+04/µL Temperature: 38.9	Recurrent (ZQ21-085D28) Parasitemia: 2.88e+04/µL Temperature: 36.5
M313	232, 258	208, 234
M383	125, 137	125, 139
TA1	168	162, 168
POLYA	150, 153	132, 138, 150
PFPK2	171, 183	171
M2490	81	81
TA109	160, 172, 184	175, 181

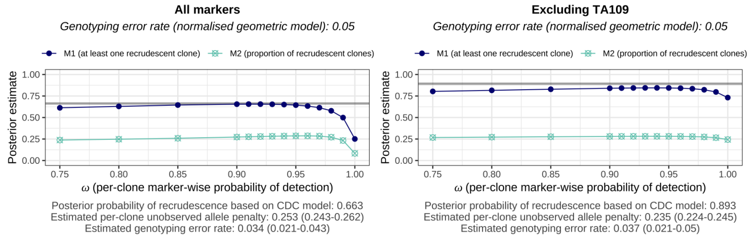

(A4)

	Baseline (LL21-094D0) Parasitemia: 1.22e+05/µL Temperature: 38.3	Recurrent (LL21-094D21) Parasitemia: 2.18e+02/µL Temperature: 35.8
M313	218, 222	258
M383	143	163
TA1	159, 165	162
POLYA	144, 150, 153, 177	150, 228
PFPK2	165, 168, 174	165
M2490	78, 81	78
TA109	163, 175	121, 175, 199

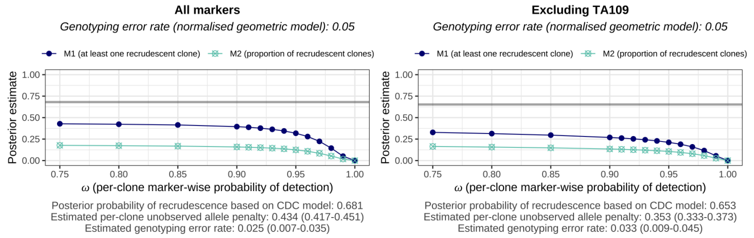

Supplementary Figure 1: Classifications of recurrences in [9] sensitive to the per-clone marker-wise probability of detection ω under PfRecur (applied to the paired baseline/recurrent samples under a marker-wise truncated multinomial model). Allele frequencies for newly-inoculated clones within each recurrence have been derived from available genotypes for baseline samples from the same study site, excluding the patient of interest, with 32 unpaired baseline samples available for Benguela (B); 26 unpaired baseline samples available for Lunda Sul (L) and 51 unpaired baseline samples available for Zaire (Z).

(A) Classification sensitive to per-clone marker-wise probability of detection

(A5)

	Baseline (BD21-053D0) Parasitemia: 9.79e+04/µL Temperature: 39.6	Recurrent (BD21-053D35) Parasitemia: 1.76e+03/µL Temperature: 36.1
M313	210	218
M383	123, 129	129
TA1	165, 168, 177, 183	
POLYA	153, 165	129, 138
PFPK2	162, 168, 180	168
M2490	75, 78, 81	
TA109	160	124, 184, 196, 199

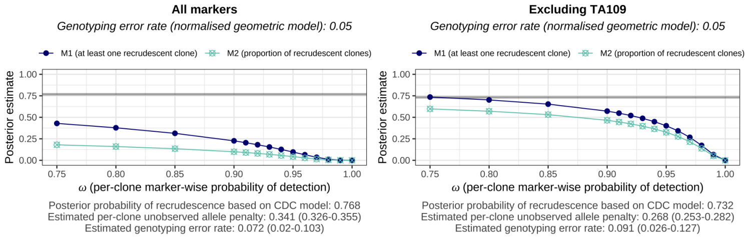

(A6)

	Baseline (ZL21-260D0) Parasitemia: 2.29e+03/µL Temperature: 37.2	Recurrent (ZL21-260D14) Parasitemia: 2.82e+02/µL Temperature: 36
M313	218, 226, 238	226, 238
M383	123, 137	123
TA1	159, 162, 165, 168, 171	141, 174
POLYA	150, 153, 171	153
PFPK2	165, 168, 189	165, 171
M2490	81, 84	81
TA109	175, 184, 199	175, 196, 199

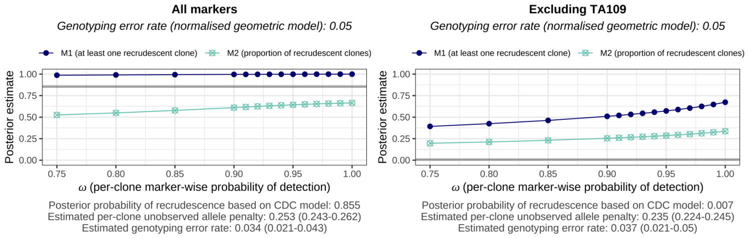

(A7)

	Baseline (ZL21-289D0) Parasitemia: 9.11e+04/µL Temperature: 38.5	Recurrent (ZL21-289D21) Parasitemia: 9.26e+02/µL Temperature: 36.1
M313	210	254
M383	125, 139	173
TA1	171, 174	159
POLYA	144, 150, 162, 177	150
PFPK2	165, 171, 177	165
M2490	78, 81, 87	78
TA109	160, 163	172, 199

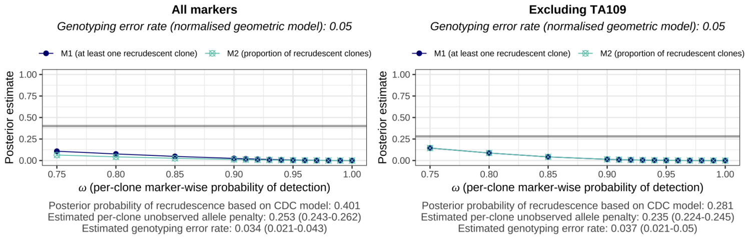

(A8)

	Baseline (LL21-077D0) Parasitemia: 2.31e+04/µL Temperature: 39	Recurrent (LL21-077D14) Parasitemia: 3.90e+01/µL Temperature: 36.1
M313	224, 236	236
M383	123, 143	143
TA1	165	
POLYA	153, 183	153
PFPK2	165, 186	186
M2490	81	78
TA109	163, 172	109, 124, 163, 184, 199

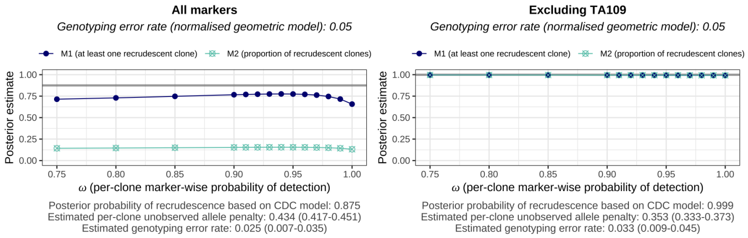

Supplementary Figure 1: Classifications of recurrences in [9] sensitive to the per-clone marker-wise probability of detection ω under PfRecur (applied to the paired baseline/recurrent samples under a marker-wise truncated multinomial model). Allele frequencies for newly-inoculated clones within each recurrence have been derived from available genotypes for baseline samples from the same study site, excluding the patient of interest, with 32 unpaired baseline samples available for Benguela (B); 26 unpaired baseline samples available for Lunda Sul (L) and 51 unpaired baseline samples available for Zaire (Z).

(B) Accommodating imperfect detection protects against possible human error

(B1)

	Baseline (ZQ21-103D0) Parasitemia: 1.59e+05/µL Temperature: 38.8	Recurrent (ZQ21-103D18) Parasitemia: 1.89e+05/µL Temperature: 36
M313	226, 238, 256	238
M383	133, 139	139
TA1	159, 165	195
POLYA	150, 156	150
PFPK2	168, 171, 186, 189, 192	171
M2490	72, 78, 81	78
TA109	160, 163, 172, 175, 184	184

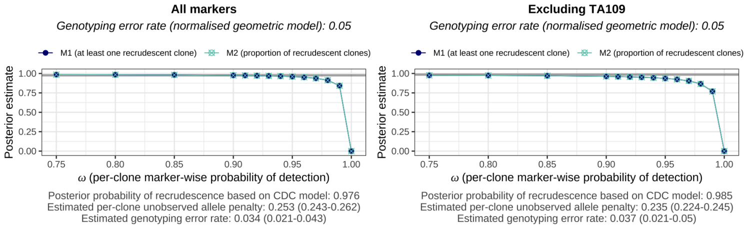

(B2)

	Baseline (ZL21-245D0) Parasitemia: 1.58e+05/µL Temperature: 37.9	Recurrent (ZL21-245D21) Parasitemia: 2.20e+04/µL Temperature: 35.6
M313	228, 234	234
M383	125, 131	151
TA1	162, 165, 183	165
POLYA	156, 159, 177	159
PFPK2	159, 165	159
M2490	78, 81	81
TA109	163, 175, 184	163

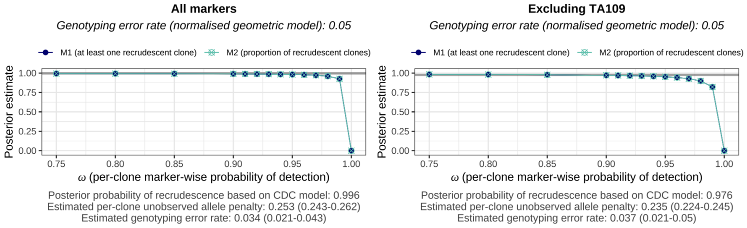

(B3)

	Baseline (LL21-010D0) Parasitemia: 4.79e+04/µL Temperature: 35.4	Recurrent (LL21-010D14) Parasitemia: 4.75e+01/µL Temperature: 36.7
M313	256	246, 284
M383	123	123
TA1	171	171
POLYA	159	111, 159
PFPK2	183	183
M2490	75	72
TA109	187	124, 187, 199

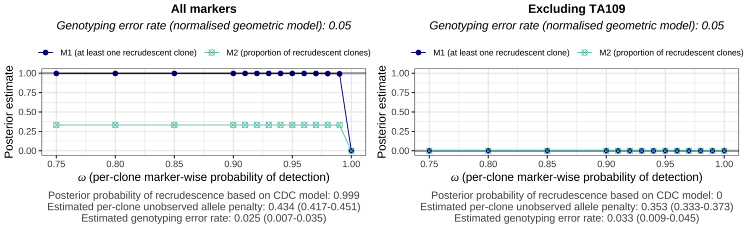

Supplementary Figure 1: Classifications of recurrences in [9] sensitive to the per-clone marker-wise probability of detection ω under PfRecur (applied to the paired baseline/recurrent samples under a marker-wise truncated multinomial model). Allele frequencies for newly-inoculated clones within each recurrence have been derived from available genotypes for baseline samples from the same study site, excluding the patient of interest, with 32 unpaired baseline samples available for Benguela (B); 26 unpaired baseline samples available for Lunda Sul (L) and 51 unpaired baseline samples available for Zaire (Z).

(C) Resolution of mixtures sensitive to per-clone marker-wise probability of detection

(C1)

	Baseline (ZL21-269D0) Parasitemia: 5.41e+04/μL Temperature: 38.9	Recurrent (ZL21-269D21) Parasitemia: 1.94e+04/μL Temperature: 37.2
M313	250	210, 250
M383	135	135
TA1	168	168
POLYA	159	150, 159
PFPK2	171, 183	171, 183
M2490	78, 81	78, 81
TA109	172	172

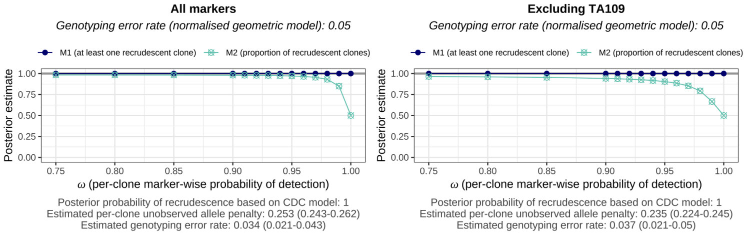

(C2)

	Baseline (ZL21-304D0) Parasitemia: 1.51e+04/μL Temperature: 37	Recurrent (ZL21-304D14) Parasitemia: 3.67e+02/μL Temperature: 36.8
M313	218, 232	218
M383	141, 151	139, 151
TA1	138, 168	138, 168
POLYA	153, 156, 162	156, 162
PFPK2	159, 162, 177, 186	159, 177
M2490	78, 81, 84	78, 81
TA109	163, 175	175, 199

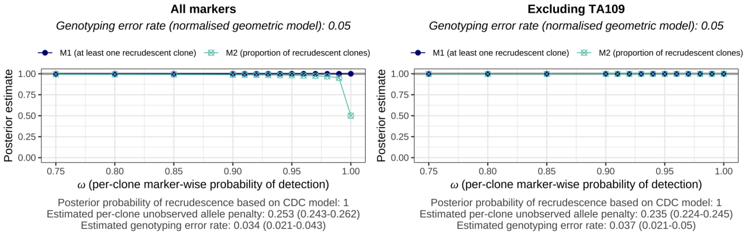

(C3)

	Baseline (LL21-054D0) Parasitemia: 2.04e+04/μL Temperature: 37.9	Recurrent (LL21-054D14) Parasitemia: 6.30e+01/μL Temperature: 38
M313	264	264
M383	131	131
TA1	171	171
POLYA	159	159
PFPK2	183, 186	186
M2490	84	
TA109	196	124, 199, 223

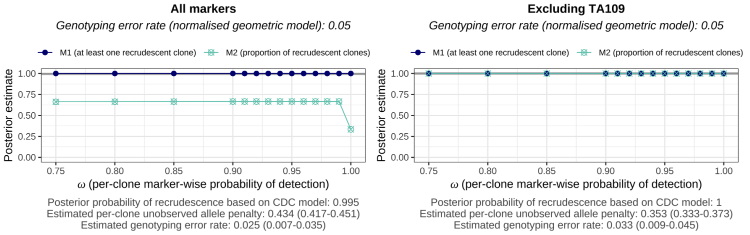

(C4)

	Baseline (ZL21-203D0) Parasitemia: 1.32e+05/μL Temperature: 37.7	Recurrent (ZL21-203D14) Parasitemia: 3.73e+02/μL Temperature: 35.8
M313	240, 246	240
M383	173	159, 173
TA1	168, 171	168
POLYA	159, 180	180
PFPK2	165, 171	171
M2490	81	81
TA109	172, 178	172, 178, 199

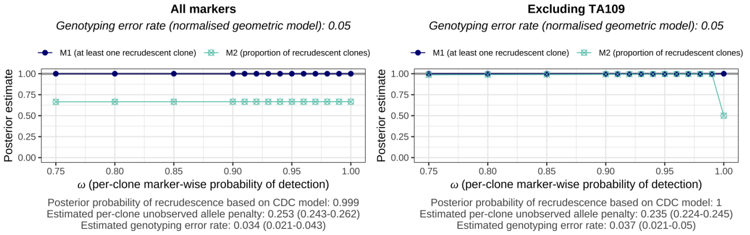

Supplementary Figure 1: Classifications of recurrences in [9] sensitive to the per-clone marker-wise probability of detection ω under PfRecur (applied to the paired baseline/recurrent samples under a marker-wise truncated multinomial model). Allele frequencies for newly-inoculated clones within each recurrence have been derived from available genotypes for baseline samples from the same study site, excluding the patient of interest, with 32 unpaired baseline samples available for Benguela (B); 26 unpaired baseline samples available for Lunda Sul (L) and 51 unpaired baseline samples available for Zaire (Z).

Classification sensitive to genotyping error

(A1)

	Baseline (ZL21-260D0) Parasitemia: 2.29e+03/μL Temperature: 37.2	Recurrent (ZL21-260D14) Parasitemia: 2.82e+02/μL Temperature: 36
M313	218, 226, 238	226, 238
M383	123, 137	123
TA1	159, 162, 165, 168, 171	141, 174
POLYA	150, 153, 171	153
PFPK2	165, 168, 189	165, 171
M2490	81, 84	81
TA109	175, 184, 199	175, 196, 199

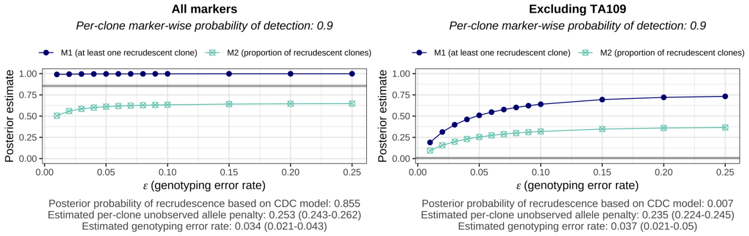

(A2)

	Baseline (ZQ21-085D0) Parasitemia: 5.01e+04/μL Temperature: 38.9	Recurrent (ZQ21-085D28) Parasitemia: 2.88e+04/μL Temperature: 36.5
M313	232, 258	208, 234
M383	125, 137	125, 139
TA1	168	162, 168
POLYA	150, 153	132, 138, 150
PFPK2	171, 183	171
M2490	81	81
TA109	160, 172, 184	175, 181

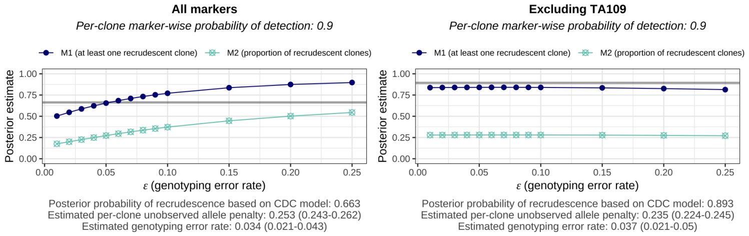

(A3)

	Baseline (ZL21-233D0) Parasitemia: 2.06e+03/μL Temperature: 37.5	Recurrent (ZL21-233D28) Parasitemia: 4.15e+03/μL Temperature: 36.4
M313	234	236
M383	147	147
TA1	177	168
POLYA	141, 144	144, 162
PFPK2	186	186
M2490	72	81
TA109	160, 175	163

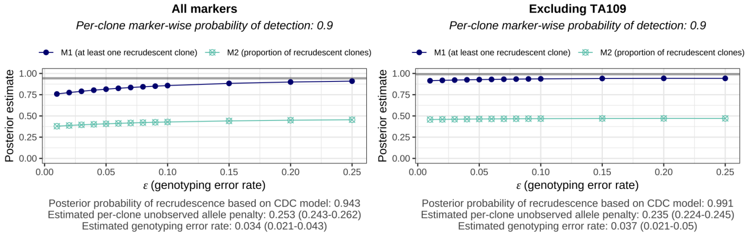

(A4)

	Baseline (LL21-061D0) Parasitemia: 5.62e+04/μL Temperature: 37.1	Recurrent (LL21-061D21) Parasitemia: 3.67e+03/μL Temperature: 36.6
M313	242, 248	242
M383	141	137
TA1	165, 168	174
POLYA	153	147
PFPK2	168, 171, 186	186
M2490	81	81
TA109	160, 172, 175	172

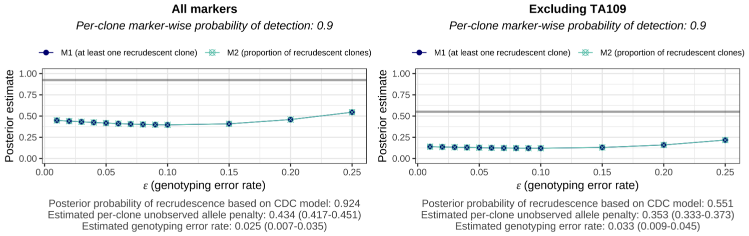

(A5)

	Baseline (BD21-053D0) Parasitemia: 9.79e+04/μL Temperature: 39.6	Recurrent (BD21-053D35) Parasitemia: 1.76e+03/μL Temperature: 36.1
M313	210	218
M383	123, 129	129
TA1	165, 168, 177, 183	
POLYA	153, 165	129, 138
PFPK2	162, 168, 180	168
M2490	75, 78, 81	
TA109	160	124, 184, 196, 199

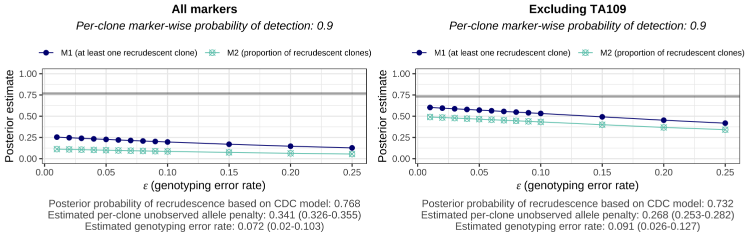

Supplementary Figure 2: Classifications of recurrences in [9] under PfRecur sensitive to the genotyping error probability ε (applied to each allele called in a baseline sample relative to the recurrent sample of interest) under the normalised geometric model (20). Allele frequencies for newly-inoculated clones within each recurrence have been derived from available genotypes for baseline samples from the same study site, excluding the patient of interest, with 32 unpaired baseline samples available for Benguela (B); 26 unpaired baseline samples available for Lunda Sul (L) and 51 unpaired baseline samples available for Zaire (Z).

Classification sensitive to genotyping error

(A6)

	Baseline (ZL21-227D0) Parasitemia: 7.69e+04/µL Temperature: 37.5	Recurrent (ZL21-227D28) Parasitemia: 1.44e+03/µL Temperature: 36
M313	230	228, 234
M383	125	123
TA1	171	168
POLYA	141, 162	156
PFPK2	171	168
M2490	72, 81	87
TA109	172, 175	172

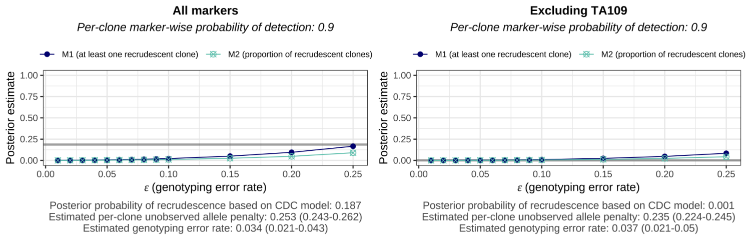

(A7)

	Baseline (ZQ21-031D0) Parasitemia: 4.48e+04/µL Temperature: 38.5	Recurrent (ZQ21-031D28) Parasitemia: 8.80e+04/µL Temperature: 36
M313	240, 254	250
M383	139	155
TA1	162, 177	165
POLYA	156, 183	159
PFPK2	159, 162	159
M2490	81	81
TA109	163, 172	172

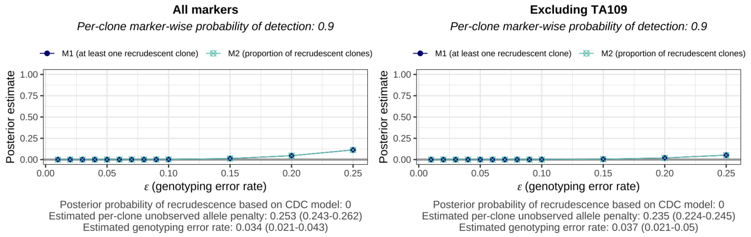

(A8)

	Baseline (LL21-077D0) Parasitemia: 2.31e+04/µL Temperature: 39	Recurrent (LL21-077D14) Parasitemia: 3.90e+01/µL Temperature: 36.1
M313	224, 236	236
M383	123, 143	143
TA1	165	
POLYA	153, 183	153
PFPK2	165, 186	186
M2490	81	78
TA109	163, 172	109, 124, 163, 184, 199

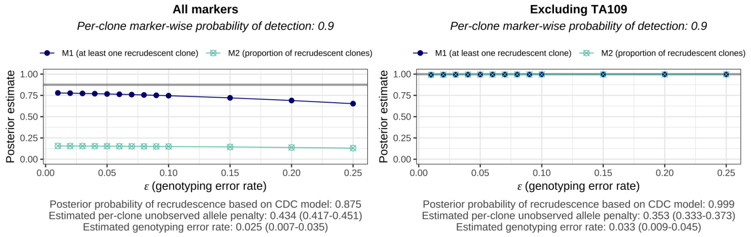

(A9)

	Baseline (LL21-094D0) Parasitemia: 1.22e+05/µL Temperature: 38.3	Recurrent (LL21-094D21) Parasitemia: 2.18e+02/µL Temperature: 35.8
M313	218, 222	258
M383	143	163
TA1	159, 165	162
POLYA	144, 150, 153, 177	150, 228
PFPK2	165, 168, 174	165
M2490	78, 81	78
TA109	163, 175	121, 175, 199

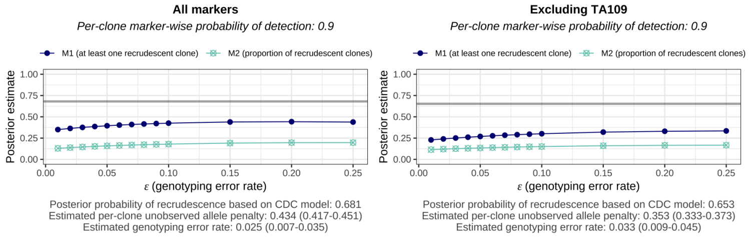

(A10)

	Baseline (LL21-086D0) Parasitemia: 1.19e+05/µL Temperature: 38.7	Recurrent (LL21-086D21) Parasitemia: 1.57e+03/µL Temperature: 36.6
M313	238	232
M383	131, 139	125, 141
TA1	177	171
POLYA	150, 165	165
PFPK2	168, 189	186
M2490	81, 84	81
TA109	163, 184	124, 175

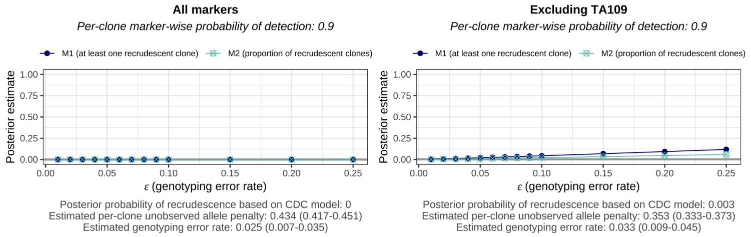

Supplementary Figure 2: Classifications of recurrences in [9] under PfRecur sensitive to the genotyping error probability ε (applied to each allele called in a baseline sample relative to the recurrent sample of interest) under the normalised geometric model (20). Allele frequencies for newly-inoculated clones within each recurrence have been derived from available genotypes for baseline samples from the same study site, excluding the patient of interest, with 32 unpaired baseline samples available for Benguela (B); 26 unpaired baseline samples available for Lunda Sul (L) and 51 unpaired baseline samples available for Zaire (Z).

3.2.2 False positive recrudescence rates

Relaxing the per-clone marker-wise probability of detection ω tends to amplify the posterior probability of recrudescence under PfRecur. However, false positive recrudescence rates across 500 permuted artificial 'not-recrudescence' datasets (obtained by generating derangements of baseline study participant labels within each study site in [9]) remain comparatively lower than the CDC model for $\varepsilon = 0.05$ and $\omega \in [0.75, 1]$ (Supplementary Figure 3). Empirical complementary cumulative distribution functions for the posterior probability of recrudescence (under the CDC model) vs metric M1 (under PfRecur) using which false positive rates are computed are shown in Supplementary Figure 4.

3.3 Comparison against CDC model

We find that the CDC model [12] typically yields elevated posterior probabilities of recrudescence for multiclonal samples with matching alleles at a subset of markers. Recurrences with differing classifications under PfRecur vs the CDC model [12] are summarised in Supplementary Figure 5.

3.3.1 Efficacy estimates

To generate efficacy estimates adjusted for classification uncertainty and right censoring, we perform the Kaplan-Meier correction used in Dimbu *et al.* [9] (as implemented in [16]): for each study arm, we perform 10,000 iterations of bootstrap resampling whereby we sample a binary reinfection or recrudescence state for each patient under a Bernoulli distribution with success parameter given by the posterior probability of recrudescence (or metric M1 under PfRecur); and then compute Kaplan-Meier estimates (including the lower 2.5% and upper 97.5% confidence interval under the `peto` method implemented in the R function `survival::survfit` (V3.3-1) [17]) for these binary infection states. Survival estimates for the last day of follow-up (28 days for AL and ASAQ; 42 days for DP and ASPY) are then averaged across these 10,000 iterations to yield an adjusted efficacy estimate and an accompanying 95% confidence interval. Efficacy estimates under PfRecur vs the CDC model (re-run on our system) are summarised in Supplementary Table 3. Efficacy estimates generated using the CDC model in Supplementary Table 3 differ from those reported in Dimbu *et al.* [9] due to the stochastic nature of the MCMC algorithm, and because early treatment failures (1 patient treated with AL, and 3 patients treated with ASAQ in Zaire) have not been taken into account in our re-analysis.

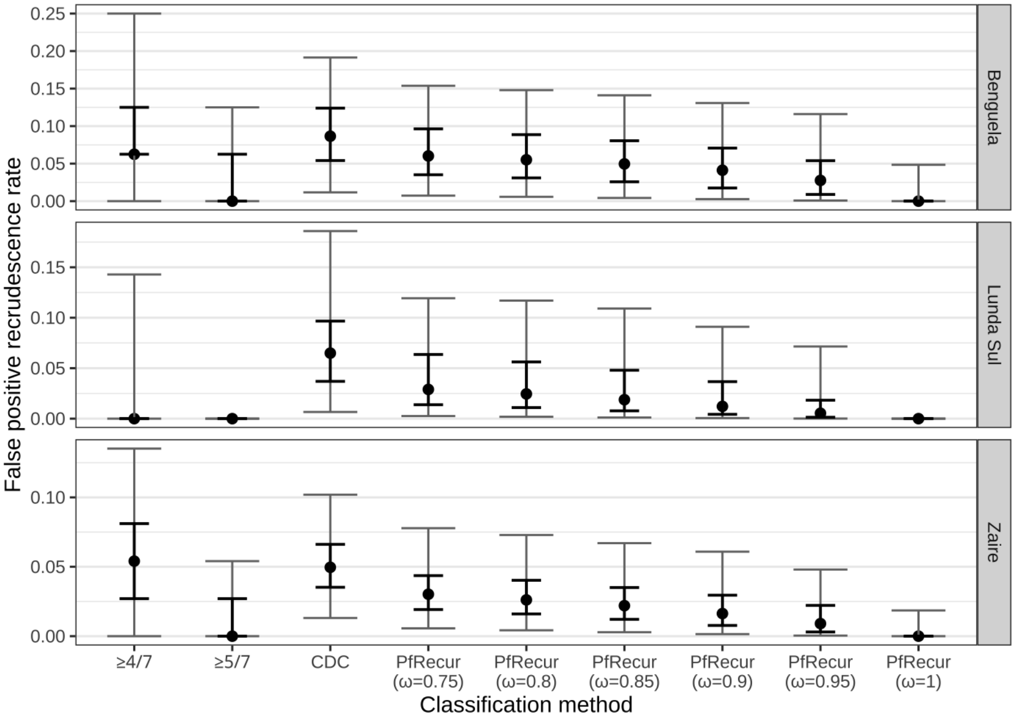

Supplementary Figure 3: Sensitivity of false positive recrudescence rates based on artificial 'non-recrudescence' data generated from [9] to the per-clone marker-wise probability of detection ω under PfRecur. We average metric M1 of PfRecur with genotyping error probability $\varepsilon = 0.05$ and per-clone marker-wise detection probability $\omega \in [0.75, 1]$. As a comparator, we show recrudescence rates based on the posterior probability of recrudescence model under the CDC model vs a match-counting approach (treating the presence of one or more shared alleles at 4 or more ($\geq 4/7$) [9, 14, 15], or 5 or more ($\geq 5/7$) markers as evidence of recrudescence, restricted to permuted pairs with ≥ 1 allele call at each of the 7 markers). Points show medians, bold error bars show the interquartile range and gray error bars indicate 95% confidence intervals across 500 permuted artificial 'not-recrudescence' datasets, each comprising 19 recurrent and 33 baseline samples from Benguela; 13 recurrent and 27 baseline samples from Lunda Sul and 38 recurrent and 52 baseline samples from Zaire [9]. For match-counting, there are 16 recurrent samples from Benguela, 7 recurrent samples from Lunda Sul and 37 recurrent samples from Zaire with at least one allele call at each of the 7 markers.

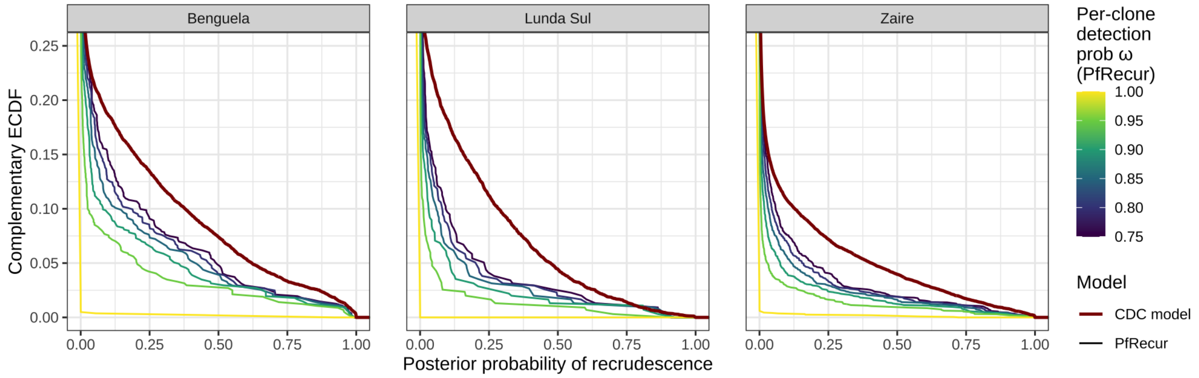

Supplementary Figure 4: Complementary empirical cumulative distribution functions (ECDFs) for the posterior probability of recrudescence (CDC model) vs metric M1 (PfRecur) aggregated over 500 **permuted artificial 'not-recrudescence' datasets generated from** [9] (each comprising 19 recurrent and 33 baseline samples from Benguela; 13 recurrent and 27 baseline samples from Lunda Sul and 38 recurrent and 52 baseline samples from Zaire).

	**Study site**	**CDC model**	**PfRecur**
**AL** (day 28)	Zaire	88.9 (82.8–95.4)	89.9 (83.9 –96.1)
	Lunda Sul	94.4 (89.9–99.0)	95.4 (91.4–99.6)
**ASAQ** (day 28)	Zaire	93.9 (89.1–98.9)	94.0 (89.3–98.9)
	Lunda Sul	100	100
**ASPY** (day 42)	Benguela	99.7 (99.2-100)	100 (100–100)
**DP** (day 42)	Benguela	98.3 (95.8–100)	98.8 (96.8–100)

Supplementary Table 3: Efficacy estimates for [9] **based on late treatment failures only, adjusted for loss to follow-up under a Kaplan-Meier model** [**12, 16**], with 95% confidence intervals. Estimates under the CDC model are based on posterior probabilities of recrudescence; estimates under PfRecur are based on metric M1 with default parameters $\omega = 0.9, \varepsilon = 0.05$. Sample sizes are detailed in Table 1 of the main text (which is adapted from Tables 1 and 3 of Dimbu *et al.* [9]).

(A) BD21-053

	PfRecur M1 >=1 clone recrudescent	PfRecur M2 Proportion recrudescent	CDC Probability recrudescence
All markers	0.227	0.1	0.768
Exc. TA109	0.572	0.465	0.732

	Day 0	Day 35
M313	210	218
M383	123, 129	129
TA1	165, 168, 177, 183	
POLYA	153, 165	129, 138
PFPK2	162, 168, 180	168
M2490	75, 78, 81	
TA109	160	124, 184, 196, 199

(B) ZL21-242

	PfRecur M1 >=1 clone recrudescent	PfRecur M2 Proportion recrudescent	CDC Probability recrudescence
All markers	0.003	0.001	0.387
Exc. TA109	0.016	0.008	0.544

	Day 0	Day 14
M313	222	240
M383	139	137
TA1	198	165
POLYA	156	153
PFPK2	168, 189	132, 168
M2490	81	87
TA109	175	124, 160, 199

(C) LL21-061

	PfRecur M1 >=1 clone recrudescent	PfRecur M2 Proportion recrudescent	CDC Probability recrudescence
All markers	0.418	0.418	0.924
Exc. TA109	0.128	0.128	0.551

	Day 0	Day 21
M313	242, 248	242
M383	141	137
TA1	165, 168	174
POLYA	153	147
PFPK2	168, 171, 186	186
M2490	81	81
TA109	160, 172, 175	172

(D) ZL21-260

	PfRecur M1 >=1 clone recrudescent	PfRecur M2 Proportion recrudescent	CDC Probability recrudescence
All markers	0.997	0.611	0.855
Exc. TA109	0.509	0.255	0.007

	Day 0	Day 14
M313	218, 226, 238	226, 238
M383	123, 137	123
TA1	159, 162, 165, 168, 171	141, 174
POLYA	150, 153, 171	153
PFPK2	165, 168, 189	165, 171
M2490	81, 84	81
TA109	175, 184, 199	175, 196, 199

(E) LL21-094

	PfRecur M1 >=1 clone recrudescent	PfRecur M2 Proportion recrudescent	CDC Probability recrudescence
All markers	0.395	0.158	0.681
Exc. TA109	0.269	0.135	0.653

	Day 0	Day 21
M313	218, 222	258
M383	143	163
TA1	159, 165	162
POLYA	144, 150, 153, 177	150, 228
PFPK2	165, 168, 174	165
M2490	78, 81	78
TA109	163, 175	121, 175, 199

(F) ZL21-289

	PfRecur M1 >=1 clone recrudescent	PfRecur M2 Proportion recrudescent	CDC Probability recrudescence
All markers	0.025	0.013	0.401
Exc. TA109	0.014	0.014	0.281

	Day 0	Day 21
M313	210	254
M383	125, 139	173
TA1	171, 174	159
POLYA	144, 150, 162, 177	150
PFPK2	165, 171, 177	165
M2490	78, 81, 87	78
TA109	160, 163	172, 199

(G) ZQ21-077

	PfRecur M1 >=1 clone recrudescent	PfRecur M2 Proportion recrudescent	CDC Probability recrudescence
All markers	1	1	0.949
Exc. TA109	0.998	0.998	0.697

	Day 0	Day 21
M313	224	224
M383	123, 137, 145	139
TA1	159, 165	165
POLYA	147, 159	159
PFPK2	171, 186	186
M2490	81	81
TA109	175, 178, 184	178

(H) LL21-038

	PfRecur M1 >=1 clone recrudescent	PfRecur M2 Proportion recrudescent	CDC Probability recrudescence
All markers	0	0	0
Exc. TA109	0	0	0.28

	Day 0	Day 28
M313	230	234
M383	123	135
TA1	177	
POLYA	162	135, 462
PFPK2	162, 171	
M2490	81	
TA109	160, 175	97, 124, 199

(I) LL21-008

	PfRecur M1 >=1 clone recrudescent	PfRecur M2 Proportion recrudescent	CDC Probability recrudescence
All markers	0.001	0	0.201
Exc. TA109	0.001	0	0.147

	Day 0	Day 21
M313	230, 252	242
M383	139	149
TA1	165	141, 159
POLYA	147	111, 156, 351
PFPK2	177	150
M2490	78	72
TA109	187	124, 133, 184, 199

(J) LL21-027

	PfRecur M1 >=1 clone recrudescent	PfRecur M2 Proportion recrudescent	CDC Probability recrudescence
All markers	0	0	0
Exc. TA109	0	0	0.18

	Day 0	Day 28
M313	218, 224, 238	
M383	123, 137	181
TA1	174	
POLYA	141, 156, 165	
PFPK2	165, 171	
M2490	78, 81	
TA109	166, 178	199

(K) ZL21-227

	PfRecur M1 >=1 clone recrudescent	PfRecur M2 Proportion recrudescent	CDC Probability recrudescence
All markers	0.007	0.003	0.187
Exc. TA109	0.003	0.002	0.001

	Day 0	Day 28
M313	230	228, 234
M383	125	123
TA1	171	168
POLYA	141, 162	156
PFPK2	171	168
M2490	72, 81	87
TA109	172, 175	172

(L) BP21-280

	PfRecur M1 >=1 clone recrudescent	PfRecur M2 Proportion recrudescent	CDC Probability recrudescence
All markers	0	0	0.171
Exc. TA109	0.001	0	0.093

	Day 0	Day 35
M313	238	220
M383	141	131
TA1	165	174, 180
POLYA	153	144, 150
PFPK2	162, 171, 204	168
M2490	81	72, 81
TA109	172, 175, 184, 187	160, 172

(M) ZQ21-046

	PfRecur M1 >=1 clone recrudescent	PfRecur M2 Proportion recrudescent	CDC Probability recrudescence
All markers	0.004	0.002	0.141
Exc. TA109	0.008	0.003	0.167

	Day 0	Day 14
M313	242	210
M383	141, 147	135
TA1	186	171, 183
POLYA	153	153, 162
PFPK2	189	156, 159, 165
M2490	81	78
TA109	160	163

(N) LL21-037

	PfRecur M1 >=1 clone recrudescent	PfRecur M2 Proportion recrudescent	CDC Probability recrudescence
All markers	0	0	0
Exc. TA109	0.002	0.001	0.14

	Day 0	Day 14
M313	236	242
M383	123	123
TA1	159, 192	168
POLYA	150	141, 177
PFPK2	171, 189	159
M2490	81	75
TA109	160, 163, 175	172, 199

(O) ZL21-233

	PfRecur M1 >=1 clone recrudescent	PfRecur M2 Proportion recrudescent	CDC Probability recrudescence
All markers	0.815	0.407	0.943
Exc. TA109	0.928	0.464	0.991

	Day 0	Day 28
M313	234	236
M383	147	147
TA1	177	168
POLYA	141, 144	144, 162
PFPK2	186	186
M2490	72	81
TA109	160, 175	163

(P) LL21-077

	PfRecur M1 >=1 clone recrudescent	PfRecur M2 Proportion recrudescent	CDC Probability recrudescence
All markers	0.767	0.153	0.875
Exc. TA109	0.995	0.995	0.999

	Day 0	Day 14
M313	224, 236	236
M383	123, 143	143
TA1	165	
POLYA	153, 183	153
PFPK2	165, 186	186
M2490	81	78
TA109	163, 172	109, 124, 163, 184, 199

(Q) BP21-245

	PfRecur M1 >=1 clone recrudescent	PfRecur M2 Proportion recrudescent	CDC Probability recrudescence
All markers	0.024	0.008	0.127
Exc. TA109	0	0	0

	Day 0	Day 28
M313	226	218
M383	125	
TA1	159	141
POLYA	159, 177	
PFPK2	171, 177, 186	216
M2490	81	
TA109	160, 175	124, 157, 199

Supplementary Figure 5: Recurrences in [9] with differing classifications under PfRecur (metrics M1 and M2) vs the CDC model [12]. For PfRecur, we take a genotyping error probability of $\varepsilon = 0.05$ under the normalised geometric model (20) (applied to each allele called in each baseline sample), and assume that the per-clone marker-wise probability of detection (applied to clones in the paired baseline/recurrent samples) is $\omega = 0.9$. Under PfRecur, allele frequencies for newly-inoculated clones within each recurrence have been derived from available genotypes for baseline samples from the same study site, excluding the patient of interest, with 32 unpaired baseline samples available for Benguela (B); 26 unpaired baseline samples available for Lunda Sul (L) and 51 unpaired baseline samples available for Zaire (Z).

4 Comparison against CDC model structure [12]

An extended comparison of the PfRecur model structure against the CDC model [12] is provided below. In brief, the CDC model [12] explicitly estimates allelic configurations using a Gibbs sampler; averages the likelihood of recrudescence across pairs of clones in a baseline and recurrent sample; and concurrently generates estimates of parametric (geometric) genotyping error probabilities and a per-clone unobserved allele penalty. In contrast, in PfRecur we average analytically over compatible allelic configurations in each sample to obtain an analytically tractable posterior; model recurrent samples as mixtures of newly-inoculated vs recrudescent clones; and treat both genotyping error and the per-clone marker-wise probability of detection for paired baseline/recurrent samples as user-specified parameters.

Supplementary Table 4: Comparison of PfRecur vs CDC model structure

**Feature**	**CDC model [12]**	**PfRecur**
Model structure	Construct joint likelihood for baseline and recurrent samples, allowing the concurrent estimation of allele frequencies and genotyping error	Construct likelihood for observed genotypes in recurrent samples only
Likelihood of reinfection	Allele-frequency based likelihood predicated on population allele frequencies estimated over all available samples, excluding recrudescent alleles	Allele-frequency based likelihood for newly-inoculated clones predicated on population allele frequencies derived from baseline samples only (excluding the paired baseline sample)
Likelihood of recrudescence	For recrudescent alleles, replace allele frequencies with a genotyping error term (probability that the allele is identical to a baseline allele)	Allele-frequency based likelihood for recrudescent clones predicated on allele frequencies largely derived from the paired baseline sample, but with allelic states for ungenotyped clones in the paired baseline sample imputed based on allele frequencies for newly-inoculated clones
Adjustment for multiple comparisons	Average likelihood of recrudescence over pairwise comparisons of baseline/recurrent clones at each locus	Likelihood averaged over all compatible allelic configurations in the recurrent sample (assume a uniform distribution across compatible allelic configurations); derived allele frequencies adjusted for allelic configurations in baseline samples

Population allele frequencies	Estimated under a multinomial-Dirichlet model based on sample allele frequencies pooled across all available baseline and recurrent samples, including unobserved alleles, but without double-counting recrudescent alleles (these are defined to be the pair of alleles at each marker in a baseline/recurrent pair that have the smallest length difference; may be repeated in a given sample); initialise sample allele frequencies by summing over the observed set of alleles for each sample (i.e., weighting each sample by its marker-wise cardinality)	Allele frequencies for newly-inoculated clones derived under a multinomial-Dirichlet model based on allele counts across baseline samples excluding the paired baseline sample, averaging over compatible allelic configurations in each relevant baseline sample (assuming perfect detection of clones) and adjusted for genotyping error; allele frequencies for recrudescent clones derived over clones in the paired baseline sample, with allelic states for undetected clones in the paired baseline sample imputed using allele frequencies for newly-inoculated clones
Genotyping error structure	Parametric (approximately geometric with respect to allele lengths); estimated under a beta-beta model in the Gibbs sampler, based on the minimal distance between the closest alleles at each locus in recurrences deemed to be recrudescences	Non-parametric; user-specified; formulated relative to the recurrent sample and applied to all baseline samples (as the probability that an allele called in the baseline sample matches an allele called in the recurrent sample); per-sample allele counts (used to derive allele frequencies) are adjusted for genotyping error
Penalising unobserved alleles	Number of hidden alleles across all samples defined to be the difference between MOI and the locus cardinality; implement a per-clone unobserved allele penalty q whenever a hidden allele lies outside the set of observed alleles; q estimated under a beta-binomial model in the Gibbs sampler	Implement a marker-wise probability ω of detecting each clone in the paired baseline and recurrent samples; ω is user-specified
Adjusting for unobserved alleles	Update one randomly chosen hidden allele in a baseline/recurrent pair per iteration; sample hidden alleles uniformly at random over the set of possible alleles at that marker; likelihood ratio for Gibbs sampler based on the convolution over frequency of each allele (true underlying allele) and genotyping error (probability true allele appears as sampled hidden allele), multiplied by the per-clone unobserved allele penalty q if relevant; if a switch is to be made, add a length variability term to sampled hidden allele (adjusted for allelic binning: based on the mean SD within allelic classes at the marker)	Impute allelic states for undetected clones in the paired baseline sample based on derived population allele frequencies for newly-inoculated clones, with a marker-wise truncated binomial model for the number of clones detected at each marker; derive the locus-wise likelihood of observed genotypes for the recurrent sample over detected clones only, with a truncated multinomial model for the number of newly-inoculated vs recrudescent clones detected at each marker

References

1. Taylor, A. R. *et al.* Resolving the cause of recurrent Plasmodium vivax malaria probabilistically. *Nature Communications* **10,** 5595 (2019).

2. Taylor, A. R. *et al.* Estimation of malaria haplotype and genotype frequencies: a statistical approach to overcome the challenge associated with multiclonal infections. *Malaria Journal* **13,** 1–11 (2014).

3. Chang, H.-H. *et al.* THE REAL McCOIL: A method for the concurrent estimation of the complexity of infection and SNP allele frequency for malaria parasites. *PLOS Computational Biology* **13,** e1005348 (2017).

4. Ju, N., Liu, J. & He, Q. SNP-Slice resolves mixed infections: Simultaneously unveiling strain haplotypes and linking them to hosts. *Bioinformatics* **40** (2024).

5. Murphy, M. & Greenhouse, B. MOIRE: a software package for the estimation of allele frequencies and effective multiplicity of infection from polyallelic data. *Bioinformatics* **40,** btae619 (2024).

6. Paschalidis, A., Watson, O. J., Aydemir, O., Verity, R. & Bailey, J. A. coiaf: directly estimating complexity of infection with allele frequencies. *PLOS Computational Biology* **19,** e1010247 (2023).

7. Taylor, A. R., Vickers, E. N. & Greenhouse, B. Review of MrsFreqPhase methods: methods designed to estimate statistically malaria parasite multiplicity of infection, relatedness, frequency and phase. *Malaria Journal* **23,** 308 (2024).

8. Gerlovina, I., Gerlovin, B., Rodríguez-Barraquer, I. & Greenhouse, B. Dcifer: an IBD-based method to calculate genetic distance between polyclonal infections. *Genetics* **222,** iyac126 (2022).

9. Dimbu, P. R. *et al.* Therapeutic response to four artemisinin-based combination therapies in Angola, 2021. *Antimicrobial Agents and Chemotherapy,* e01525–23 (2024).

10. Hong, Y. On computing the distribution function for the Poisson binomial distribution. *Computational Statistics & Data Analysis* **59,** 41–51 (2013).

11. Olivella, S. & Shiraito, Y. *poisbinom: A Faster Implementation of the Poisson-Binomial Distribution* R package version 1.0.1 (2017). `https://CRAN.R-project.org/package=poisbinom`.

12. Plucinski, M. M., Morton, L., Bushman, M., Dimbu, P. R. & Udhayakumar, V. Robust algorithm for systematic classification of malaria late treatment failures as recrudescence or reinfection using microsatellite genotyping. *Antimicrobial Agents and Chemotherapy* **59,** 6096–6100 (2015).

13. Taylor, A. R., Jacob, P. E., Neafsey, D. E. & Buckee, C. O. Estimating relatedness between malaria parasites. *Genetics* **212,** 1337–1351 (2019).

14. Jones, S., Plucinski, M., Kay, K., Hodel, E. M. & Hastings, I. M. A computer modelling approach to evaluate the accuracy of microsatellite markers for classification of recurrent infections during routine monitoring of antimalarial drug efficacy. *Antimicrobial Agents and Chemotherapy* **64,** 10–1128 (2020).

15. Plucinski, M. M. & Barratt, J. L. Nonparametric Binary Classification to Distinguish Closely Related versus Unrelated Plasmodium falciparum Parasites. *The American Journal of Tropical Medicine and Hygiene* **104,** 1830 (2021).

16. Plucinski, M. *AngolaTES2021* `https://github.com/MateuszPlucinski/AngolaTES2021`. 2024.

17. Terry M. Therneau & Patricia M. Grambsch. *Modeling Survival Data: Extending the Cox Model* ISBN: 0-387-98784-3 (Springer, New York, 2000).
