# Supplementary material for: Probabilistic classification of late treatment failure in uncomplicated falciparum malaria
Source: Nat Commun. 2025 Nov 10;16:9880. doi: 10.1038/s41467-025-64830-z (PMC12603158; doi:10.1038/s41467-025-64830-z)
Supplement: Supplementary file 3 — Reporting Summary [file 41467_2025_64830_MOESM3_ESM.pdf]

nature portfolio

Corresponding author(s): Somya Mehra

Last updated by author(s): 2025/08/27

Reporting Summary

Nature Portfolio wishes to improve the reproducibility of the work that we publish. This form provides structure for consistency and transparency in reporting. For further information on Nature Portfolio policies, see our Editorial Policies and the Editorial Policy Checklist.

Statistics

For all statistical analyses, confirm that the following items are present in the figure legend, table legend, main text, or Methods section.

n/a	Confirmed	
☐	☒	The exact sample size (*n*) for each experimental group/condition, given as a discrete number and unit of measurement
☐	☒	A statement on whether measurements were taken from distinct samples or whether the same sample was measured repeatedly
☐	☒	The statistical test(s) used AND whether they are one- or two-sided<br>*Only common tests should be described solely by name; describe more complex techniques in the Methods section.*
☐	☒	A description of all covariates tested
☐	☒	A description of any assumptions or corrections, such as tests of normality and adjustment for multiple comparisons
☐	☒	A full description of the statistical parameters including central tendency (e.g. means) or other basic estimates (e.g. regression coefficient) AND variation (e.g. standard deviation) or associated estimates of uncertainty (e.g. confidence intervals)
☐	☒	For null hypothesis testing, the test statistic (e.g. *F*, *t*, *r*) with confidence intervals, effect sizes, degrees of freedom and *P* value noted<br>*Give P values as exact values whenever suitable.*
☐	☒	For Bayesian analysis, information on the choice of priors and Markov chain Monte Carlo settings
☒	☐	For hierarchical and complex designs, identification of the appropriate level for tests and full reporting of outcomes
☒	☐	Estimates of effect sizes (e.g. Cohen's *d*, Pearson's *r*), indicating how they were calculated

Our web collection on statistics for biologists contains articles on many of the points above.

Software and code

Policy information about availability of computer code

Data collection

No software was used for data collection.

Data analysis

The PfRecur framework has been implemented in an eponymous R package, openly available in a GitHub repository:
https://github.com/somyamehra/PfRecur

The version of PfRecur (v2) used in this manuscript has been linked to Zenodo:
https://doi.org/10.5281/zenodo.16965130

The PfRecur package largely relies on base R functionality, with external dependencies on the functions copula::Stirling2, copula::Stirling1, PDQutils::cumulant2moment, PDQutils::moment2cumulant, poisbinom::dpoisbinom and VGAM::dbetabinom.ab. We have tested the package with copula V1.1-1, PDQutils V0.1.6, poisbinom V1.0.1 and VGAM V1.1-9 and R V4.2.1.

We have re-run code provided openly by Dr Mateusz Plucinski and colleagues (with very minor input/output modifications), which implements the model detailed in Plucinski et al (2015) [doi: 10.1128/AAC.00072-15] and is available in a GitHub repository:
https://github.com/MateuszPlucinski/AngolaTES2021

For completeness, all code and data relevant to this study (including the data of Dimbu et al (2024) [doi: 10.1128/aac.01525-23] and the implementation of Plucinski et al (2015) [doi: 10.1128/AAC.00072-15]) have been collated in a GitHub repository:
https://github.com/somyamehra/PfTreatmentFailure

To perform statistical analyses, we have also used the R functions PMCMRplus::jonckheereTest (V1.9.6), ggplot2::geom_smooth (V3.5.1), stats::lm (V4.2.1) and survival::survfit (V3.3-1).

For manuscripts utilizing custom algorithms or software that are central to the research but not yet described in published literature, software must be made available to editors and reviewers. We strongly encourage code deposition in a community repository (e.g. GitHub). See the Nature Portfolio guidelines for submitting code & software for further information.

Data

Policy information about availability of data

All manuscripts must include a data availability statement. This statement should provide the following information, where applicable:

- Accession codes, unique identifiers, or web links for publicly available datasets
- A description of any restrictions on data availability
- For clinical datasets or third party data, please ensure that the statement adheres to our policy

This study uses open access data that has previously been published by Dimbu et al (2024) [doi: 10.1128/aac.01525-2]. Parasite densities and clinical metadata have been retrieved from Supplemental Table S4 of Dimbu et al (2024) [doi: 10.1128/aac.01525-23], while genotypic data have been retrieved from an accompanying GitHub repository: https://github.com/MateuszPlucinski/AngolaTES2021.

Research involving human participants, their data, or biological material

Policy information about studies with human participants or human data. See also policy information about sex, gender (identity/presentation), and sexual orientation and race, ethnicity and racism.

Reporting on sex and gender	N/A: we have re-analysed open access data from a therapeutic efficacy study conducted by Dimbu et al (2024) [doi: 10.1128/aac.01525-2]
Reporting on race, ethnicity, or other socially relevant groupings	N/A: we have re-analysed open access data from a therapeutic efficacy study conducted by Dimbu et al (2024) [doi: 10.1128/aac.01525-2]
Population characteristics	N/A: we have re-analysed open access data from a therapeutic efficacy study conducted by Dimbu et al (2024) [doi: 10.1128/aac.01525-2]
Recruitment	N/A: we have re-analysed open access data from a therapeutic efficacy study conducted by Dimbu et al (2024) [doi: 10.1128/aac.01525-2]
Ethics oversight	N/A: we have re-analysed open access data from a therapeutic efficacy study conducted by Dimbu et al (2024) [doi: 10.1128/aac.01525-2]

Note that full information on the approval of the study protocol must also be provided in the manuscript.

Field-specific reporting

Please select the one below that is the best fit for your research. If you are not sure, read the appropriate sections before making your selection.

☒ Life sciences ☐ Behavioural & social sciences ☐ Ecological, evolutionary & environmental sciences

For a reference copy of the document with all sections, see nature.com/documents/nr-reporting-summary-flat.pdf

Life sciences study design

All studies must disclose on these points even when the disclosure is negative.

Sample size	We have re-analysed open access data from a therapeutic efficacy study conducted by Dimbu et al (2024) [doi: 10.1128/aac.01525-2]; sample sizes were determined in the original study.
Data exclusions	We have re-analysed open access data from a therapeutic efficacy study conducted by Dimbu et al (2024) [doi: 10.1128/aac.01525-2]. We have considered all available genotypic data, comprising unphased genotypes across 7 microsatellite markers for 182 blood samples (encompassing 70 of 71 detected late treatment failures; the 70 corresponding baseline infections; and 42 additional baseline infections), retrieved from an accompanying GitHub repository (https://github.com/MateuszPlucinski/AngolaTES2021). We have also analysed parasitemia reported for these 182 blood samples (retrieved from Supplemental Table S4 of Dimbu et al (2024) [doi: 10.1128/aac.01525-23]).
Replication	We have re-analysed open access data from a therapeutic efficacy study conducted by Dimbu et al (2024) [doi: 10.1128/aac.01525-2]; our study does not include any laboratory experiments. The focus of our study is the probabilistic classification of late treatment failure. In the original study of Dimbu et al (2024), late treatment failures were classified using a 4/7 match-counting approach and the Bayesian CDC model developed Plucinski et al (2015) [doi: 10.1128/AAC.00072-15]. In the present study, we have re-run code provided openly by Plucinksi and colleagues (available in the GitHub repository https://github.com/MateuszPlucinski/AngolaTES2021) with 100,000 iterations for the Gibbs sampler; posterior probabilities of recrudescence based on all 7 microsatellite markers may differ from those reported in Dimbu et al (2024) due to the stochastic nature of the MCMC algorithm. Efficacy estimates generated using the CDC model in Supplementary Table 3 differ from those reported in Dimbu et al (2024) due to the stochastic nature of the MCMC algorithm, and because early treatment failures (1 patient treated with AL and 3 patients treated with ASAQ in Zaire) have not been taken into account in our re-analysis (genotypic data do not appear to be available for these early treatment failures).

Randomization	We have re-analysed open access data from a therapeutic efficacy study conducted by Dimbu et al (2024) [doi: 10.1128/aac.01525-2]. In line with the original study, we have stratified samples by study site when analysing genotypes (to account for differences in population structure). This applies to the classification of individual recurrences (e.g. allele frequencies for newly-inoculated clones are derived from genotypes for all baseline samples with available genotypic data from the same study site) and the estimation of false recrudescence rates (e.g. artificial 'not-recrudescence' datasets have been generated using random derangements of baseline study participants labels within each study site, using all baseline samples with available genotypic data).
Blinding	We have re-analysed open access data from a therapeutic efficacy study conducted by Dimbu et al (2024) [doi: 10.1128/aac.01525-2]; blinding was thus neither possible, nor relevant to our study.

Reporting for specific materials, systems and methods

We require information from authors about some types of materials, experimental systems and methods used in many studies. Here, indicate whether each material, system or method listed is relevant to your study. If you are not sure if a list item applies to your research, read the appropriate section before selecting a response.

Materials & experimental systems

n/a	Involved in the study	
☒	☐	Antibodies
☒	☐	Eukaryotic cell lines
☒	☐	Palaeontology and archaeology
☒	☐	Animals and other organisms
☒	☐	Clinical data
☒	☐	Dual use research of concern
☒	☐	Plants

Methods

n/a	Involved in the study	
☒	☐	ChIP-seq
☒	☐	Flow cytometry
☒	☐	MRI-based neuroimaging

Plants

Seed stocks	*Report on the source of all seed stocks or other plant material used. If applicable, state the seed stock centre and catalogue number. If plant specimens were collected from the field, describe the collection location, date and sampling procedures.*
Novel plant genotypes	*Describe the methods by which all novel plant genotypes were produced. This includes those generated by transgenic approaches, gene editing, chemical/radiation-based mutagenesis and hybridization. For transgenic lines, describe the transformation method, the number of independent lines analyzed and the generation upon which experiments were performed. For gene-edited lines, describe the editor used, the endogenous sequence targeted for editing, the targeting guide RNA sequence (if applicable) and how the editor was applied.*
Authentication	*Describe any authentication procedures for each seed stock used or novel genotype generated. Describe any experiments used to assess the effect of a mutation and, where applicable, how potential secondary effects (e.g. second site T-DNA insertions, mosiacism, off-target gene editing) were examined.*
